# Co-STARs combine the advantages of chimeric antigen and T-cell receptors for the treatment of tumors with low antigen densities

**DOI:** 10.1126/scitranslmed.adg7123

**Published:** 2024-07-10

**Authors:** Brian J. Mog, Nikita Marcou, Sarah R. DiNapoli, Alexander H. Pearlman, Tushar D. Nichakawade, Michael S. Hwang, Jacqueline Douglass, Emily Han-Chung Hsiue, Stephanie Glavaris, Katharine M. Wright, Maximilian F. Konig, Suman Paul, Nicolas Wyhs, Jiaxin Ge, Michelle S. Miller, P. Aitana Azurmendi, Evangeline Watson, Drew M. Pardoll, Sandra B. Gabelli, Chetan Bettegowda, Nickolas Papadopoulos, Kenneth W. Kinzler, Bert Vogelstein, Shibin Zhou

**Affiliations:** 1 Ludwig Center, Sidney Kimmel Comprehensive Cancer Center, The Johns Hopkins University School of Medicine, Baltimore, MD 21287, USA; 2 Howard Hughes Medical Institute, Chevy Chase, MD 20815, USA; 3 Lustgarten Pancreatic Cancer Research Laboratory, Sidney Kimmel Comprehensive Cancer Center, The Johns Hopkins University School of Medicine, Baltimore, MD 21287, USA; 4 Department of Biomedical Engineering, Johns Hopkins University, Baltimore, MD 21218, USA; 5 Department of Chemical and Biomolecular Engineering, Johns Hopkins University, Baltimore, MD 21218, USA; 6 Institute for NanoBioTechnology, Johns Hopkins University, 3400 N Charles St, Baltimore, MD 21218, USA; 7 Department of Oncology, The Johns Hopkins University School of Medicine, Baltimore, MD 21287, USA; 8 Department of Biophysics and Biophysical Chemistry, The Johns Hopkins University School of Medicine, Baltimore, MD 21205, USA; 9 Bloomberg~Kimmel Institute for Cancer Immunotherapy, Sidney Kimmel Comprehensive Cancer Center, Baltimore, MD 21287, USA; 10 Division of Rheumatology, Department of Medicine, The Johns Hopkins University School of Medicine, Baltimore, MD 21224, USA; 11 Department of Medicine, The Johns Hopkins University School of Medicine, Baltimore, MD 21205, USA; 12 Department of Neurosurgery, The Johns Hopkins University School of Medicine, MD 21205, USA; 13 Department of Pathology, The Johns Hopkins University School of Medicine, Baltimore, MD 21205, USA

## Abstract

Two types of engineered T cells have been successfully used to treat patients with cancer, one with an antigen recognition domain derived from antibodies (chimeric antigen receptors, CARs) and the other derived from T-cell receptors (TCRs). CARs employ high-affinity antigen binding domains and co-stimulatory domains to augment T-cell activation but can only react against target cells with relatively high amounts of antigen. TCRs have a much lower affinity for their antigens but can react against target cells displaying only a few antigen molecules. Here we describe a new type of receptor, called a Co-STAR (for *C*o-stimulatory *S*ynthetic *T*-cell receptor and *A*ntigen *R*eceptor), that combines aspects of both CARs and TCRs. In Co-STARs, the antigen-recognizing components of TCRs are replaced by high-affinity antibody fragments and co-stimulation is provided by two modules that drive NFκB signaling (MyD88 and CD40). Using a TCR-mimic antibody fragment that targets a recurrent p53 neoantigen presented in a common human leukocyte antigen (HLA) allele, we demonstrate that T cells equipped with Co-STARs can kill cancer cells bearing low densities of antigen better than T cells engineered with conventional CARs and patient-derived TCRs in vitro. In mouse models, we show that Co-STARs mediate more robust T-cell expansion and more durable tumor regressions than TCRs similarly modified with MyD88 and CD40 co-stimulation. Our data suggest that Co-STARs may have utility for other peptide-HLA antigens in cancer as well as other targets where antigen density may limit the efficacy of engineered T cells.

## One-Sentence Summary

Co-STARs enable sustained anti-tumor activity against a low-density neoantigen in mouse tumors.

T cell-based therapeutics are one of the most promising approaches to treat advanced cancers and are now the subject of intense research. Naturally-occurring, tumor-targeted T cells from patients with cancer, and more recently T cells engineered to express the T cell receptors (TCRs) from patients’ T cells, have been shown to induce remissions in a subset of patients with solid tumors that have failed multiple previous therapies (1–5). T cells modified with chimeric antigen receptors (CARs) have produced substantial improvements in clinical outcomes for patients with leukemias, lymphomas, and multiple myeloma and are now being developed for other tumor types (6–9). However, many challenges remain. For example, the lack of persistence of engineered T cells following their administration is often the cause of eventual treatment failure (10–20). Another challenge is that the treatment of common solid tumors with engineered T cells has yet to come to fruition in the clinic (21–25). The identification of suitable tumor-specific antigens on solid tumors for T cells to target will likely be critical for future progress in this area.

As with any powerful cancer therapeutic agent, success depends on specificity. For instance, when CAR T cells have been directed against a target that is also expressed in normal tissues, life-threatening on-target, off-tumor toxicities have occurred (26–33). At present, the clinically-approved T-cell-based therapeutic agents are not tumor-specific – they can be used because they target antigens on normal B cells or plasma cells that are dispensable for patient survival. Antigens derived from mutant oncogenes or tumor suppressor genes can be used to unequivocally distinguish cancer cells from all normal cells. Although most of these genes produce intracellular proteins, proteolytically processed peptides derived from these mutant genes can be presented on the tumor cell surface by binding to human leukocyte antigen (HLA) molecules to form mutation-associated neoantigens (MANAs) that can serve as targets for therapeutic T cells (34–36). In addition to conventional TCRs, a unique class of TCR-mimic antibodies called MANAbodies has been developed to target MANAs bound to patients’ HLA molecules (37–40). MANAbodies have much higher affinities than TCRs (nM vs. μM) and are much easier to discover and improve than TCRs using techniques such as phage display, yeast display, and ribosome display (41, 42). Despite the promise of exquisite cancer specificity, MANAbody equipped CAR T cells must overcome the challenge of targeting peptide-HLA (pHLA) complexes that are found at extremely low densities on the surface of cancer cells – often fewer than 10 copies per cell – well below the activation threshold of conventional CARs (38, 39, 43–46).

The TCR is the gold standard for antigen sensitivity because it can activate T cells by engaging as few as a single pHLA antigen on a targeted cell (44, 47–49). The higher antigen sensitivity of TCRs compared to CARs is thought to be due, in part, to the multi-subunit architecture of the TCR complex: the low affinity antigen recognition domains of the TCR α and β chains are non-covalently associated with the CD3 ε, γ, δ, and ζ chains which contain the intracellular ITAM domains responsible for TCR signal propagation (44, 49, 50). Indeed, the antigen sensitivity of antibody binding domains has been improved by transplanting antibody domains from the CAR scaffold to the TCR complex to create hybrid TCR-like CARs, either as single-chain variable fragments (scFvs) attached to CD3 and TCR subunits (T-cell receptor fusion constructs, TRuCs) or by replacing TCR variable domains with antibody variable domains (synthetic T-cell receptor and antigen receptor, STAR, or HLA-independnent T-cell receptor, HIT) (51–53). Hybrid TCR-like CARs have not been tested against cells bearing antigen densities as low as those observed in cancers displaying peptides derived from cancer driver genes (one or two pHLA complexes per cell) (34, 39, 53). A recent study testing TCR-mimic antibody receptors in peptide pulsed cells demonstrated that TCRs and STAR receptor formats have similar antigen sensitivities, but it is not clear whether antibody derived domains transplanted into the TCR complex can produce robust anti-tumor activity against the much lower densities of endogenously processed and presented pHLA antigens found on cancer cells (50). In the present work, we describe the development of high-affinity antibody-expressing T cells that can generate sustained T-cell activity against cancer cells expressing low-antigen densities.

## Results

The antigen targeted in the current study is a peptide-HLA complex (pHLA) composed of a peptide containing the *TP53* R175H mutation bound to HLA-A*02:01. The *TP53* R175H mutation is the most common mutation in *TP53*, and HLA-A*02:01 is the most common HLA allele in Western populations (54, 55). However, the p53^R175H^/HLA-A*02:01 neoantigen is expressed on the surface of cancer cells at very low densities (an estimated 1.3 to 2.4 molecules per cell) (39). Thus, the p53^R175H^/HLA-A*02:01 neoantigen, hereinafter termed p53RH antigen, is a suitable target for the development of new T-cell therapeutic agents with improved potency against clinically relevant neoantigens expressed at low densities on target cells.

### Comparing conventional CARs and TCRs at low antigen densities

**Fig. 1.**
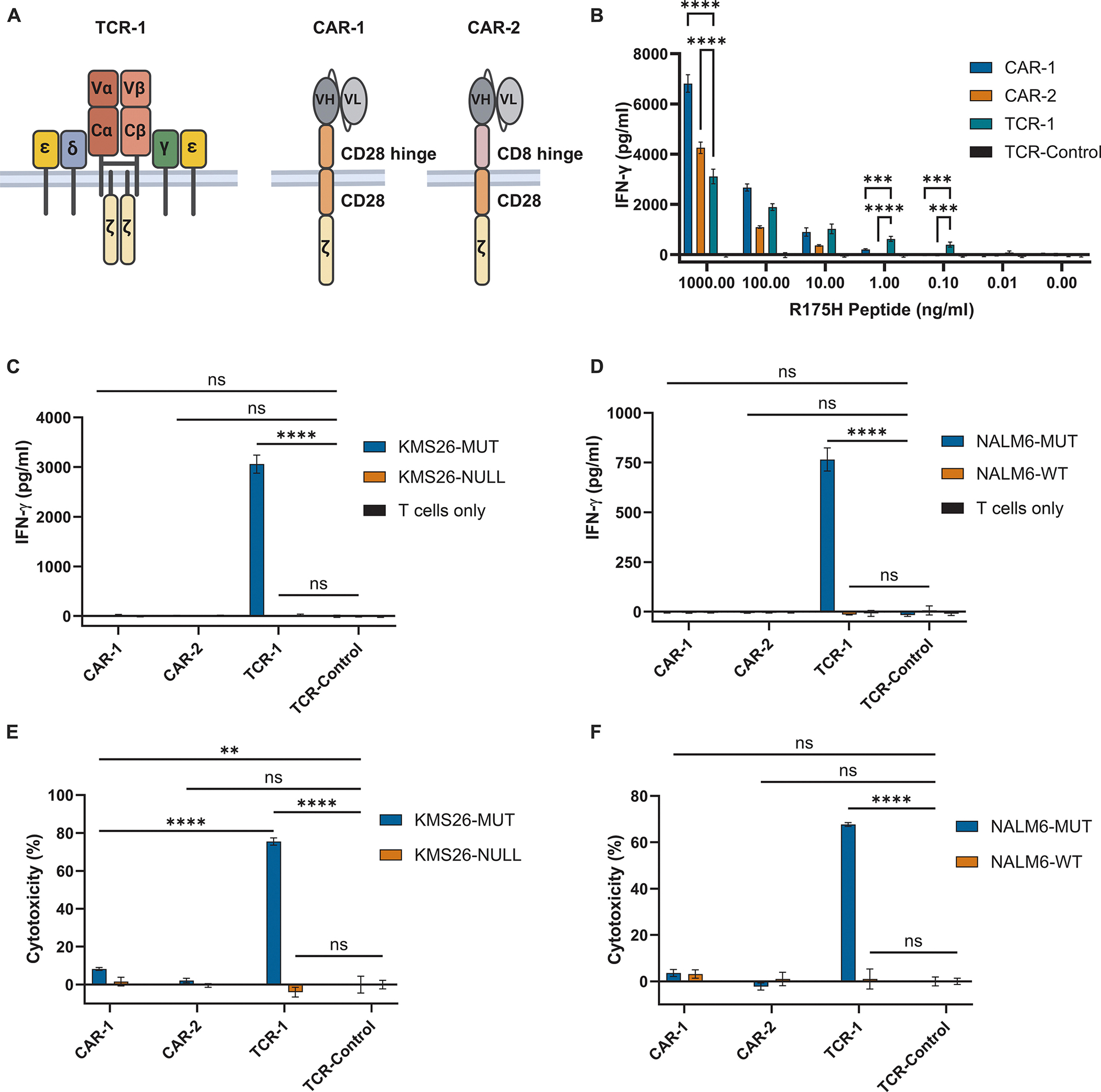
Potencies of conventional CARs and TCRs vary with antigen density. (A) Diagrams of TCR-1, CAR-1, and CAR-2. CAR-1 employs a CD28 hinge, whereas CAR-2 employs a CD8α hinge. Both CARs use a CD28 transmembrane domain and intracellular signaling domain followed by a CD3ζ intracellular domain. Vα, Vβ, Cα and Cβ denote TCR variable α, variable β, constant α and constant β chains, respectively; ε, δ, γ and ζ denote the ε, δ, γ and ζ CD3 subunits, respectively; VH and VL, variable heavy and light chains of scFv, respectively. (B) T2 cells pulsed with decreasing concentrations of the p53RH peptide (HMTEVVRHC) were incubated with modified T cells at an E:T ratio of 1:5 for 24 hrs. Conditioned supernatant was assessed for IFN-γ by ELISA. (C) Modified T cells were cultured with the KMS26 isogenic cell set at an E:T ratio of 1:5 for 20 hrs. Conditioned supernatant was assayed for IFN-γ by ELISA. (D) Modified T cells were cultured with the NALM6 isogenic cell set exactly as described in C. (E) Modified T cells were cultured with the KMS26 isogenic cell set at an E:T ratio of 1:5 for 20 hrs. Cytotoxicity was quantified by bioluminescence since the target cancer cell lines were modified to express luciferase. (F) Modified T cells were cultured with the NALM6 isogenic cell set exactly as described in E. All data are shown as means ± SD of three technical replicates, except for the T cells only conditions, which represent two technical replicates. Number of repeated experiments, N = 2, with n = 2 different donors. ****P < 0.0001, ***P < 0.001, ns (not significant) by two-way ANOVA with Tukey’s multiple comparison test.

H2 is a single-chain variable fragment (scFv) directed against the p53RH antigen with an affinity of 86 nM when measured in a T cell-engaging single-chain diabody format (39). The H2-scFv was grafted onto a conventional second-generation CAR containing CD28 hinge, transmembrane, and co-stimulatory domains linked to a CD3ζ intracellular domain to create CAR-1 (Fig. 1A). H2 was also grafted onto another second-generation CAR format that used a CD8α hinge domain instead of a CD28 hinge to create CAR-2 (Fig. 1A). A TCR construct targeting the p53RH antigen was also generated (TCR-1 in Fig. 1A). TCR-1 was based on the patient-derived AV6/BV11 TCR demonstrated to have pre-clinical as well as clinical utility, with an affinity of 3.5 μM (56, 57). From here we will refer to these as Tier 1 constructs.

To compare different constructs in a way that minimized confounding factors that could impact the results, we used CRISPR-based homology-directed repair (HDR) strategies to introduce all constructs into the T-cell receptor α constant (*TRAC*) locus of primary human T cells (fig. S1A) (58). In all cases unless otherwise noted, the expression was driven by an EF1α promoter based on the previous discovery that this promoter increases the expression of a transgenic TCR over that of the endogenous *TRAC* promoter (59). Concomitantly, the *TRBC* loci were inactivated to avoid competition from endogenous TCR chains in the engineered cells (fig. S1A). Thus, the endogenous TCRs were not expressed in the engineered cells. The absence of endogenous TCR expression also prevented allogeneic reactivity of the engineered T cells, which may complicate interpretation of the data from long-term cell culture or animal models.

Primary human T cells engineered with Tier 1 constructs CAR-1, CAR-2, or TCR-1 all bound the p53RH antigen in both the CD4 and CD8 T-cell subsets when measured by flow cytometry (fig. S1B). Control T cells (TCR-Control), in which the *TRAC* and *TRBC* loci were inactivated but no CAR or TCR components were introduced, were included in all experiments (fig. S1B). To assess their signaling capacity, the cells were co-cultured with T2 cells that express low amounts of peptide-bound HLA-A*02:01 on the cell surface due to a deficiency of the transporter associated with antigen processing (TAP) gene, and therefore preferentially present exogenously provided peptides. T2 cells were first incubated with various concentrations of a synthetic nine amino acid peptide containing the *TP53* R175H mutation to generate different p53RH antigen densities and were then co-cultured with the modified T cells described above (Fig. 1A). At very high concentrations of antigen, CAR-1 and CAR-2 T cells were activated as much as or more than TCR-1 T cells, as assessed by secretion of the cytokine interferon-γ (IFN-γ) (*P* < 0.0001; Fig. 1B). However, at lower densities of presented antigen, TCR-1 cells were activated more than CAR-1 or CAR-2 cells (P < 0.001; Fig. 1B). These data confirm previous observations that TCRs can signal much more effectively than CARs against low-density antigens (44, 49, 50).

We next determined the potency of the Tier 1 T cells when co-cultured with target cells that endogenously process and present the p53RH antigen. KMS26-MUT cells endogenously express HLA-A*02:01 and a naturally-occurring mutant *TP53* R175H allele. CRISPR was used to create a paired line, KMS26-NULL, in which both alleles of the endogenous *TP53* gene were disrupted (39). NALM6-WT cells express HLA-A*02:01 and wildtype (WT) *TP53* alleles. CRISPR was used to create NALM6-MUT cells in which the WT *TP53* allele was replaced with a mutant *TP53* R175H allele (fig. S2). The average numbers of the p53RH antigen on the cell surface were 2.4 and 1.3 molecules per cell on KMS26-MUT and NALM6-MUT cells, respectively, as measured by mass spectrometry (table S1). After co-culture with the KMS26-MUT cells, TCR-1 T cells, but not CAR-1 or CAR-2 T cells, secreted IFN-γ (*P* < 0.0001; Fig. 1C). The same pattern of activation was observed after co-culture of TCR-1, CAR-1, and CAR-2 T cells with NALM6-MUT cells (*P* < 0.0001; Fig. 1D). The KMS26-NULL and NALM6-WT cancer cells served as specificity controls in these experiments (Fig. 1, C and D).

To determine whether the cytokine activation was accompanied by killing of target cells, the T cells were co-cultured for 20 hours with the same p53RH-expressing cancer cells. Substantial cytotoxicity compared to TCR-Control T cells was observed with TCR-1 T cells for both KMS26-MUT and NALM6-MUT target cells (*P* < 0.0001; Fig. 1, E and F). No cytotoxicity was observed for CAR-2 T cells co-cultured with either target cell type and CAR-1 T cells co-cultured with the NALM6-MUT cells (*P* > 0.05; Fig. 1, E and F). CAR-1 T cells demonstrated detectable cytotoxicity against the KMS26-MUT cell line (P < 0.01) but 9-fold less than TCR-1 T cells (P < 0.0001; Fig. 1, E and F). Specificity for the p53RH antigen was documented using isogenic KMS26-NULL and NALM6-WT cells (*P* > 0.05; Fig. 1, E and F). These results confirm the superior capacity of TCRs over conventional CARs to activate T cells in response to low-density antigens as measured by IFN-γ secretion and cytotoxicity.

### Hybrid TRuC T cells have improved antigen sensitivity

**Fig. 2.**
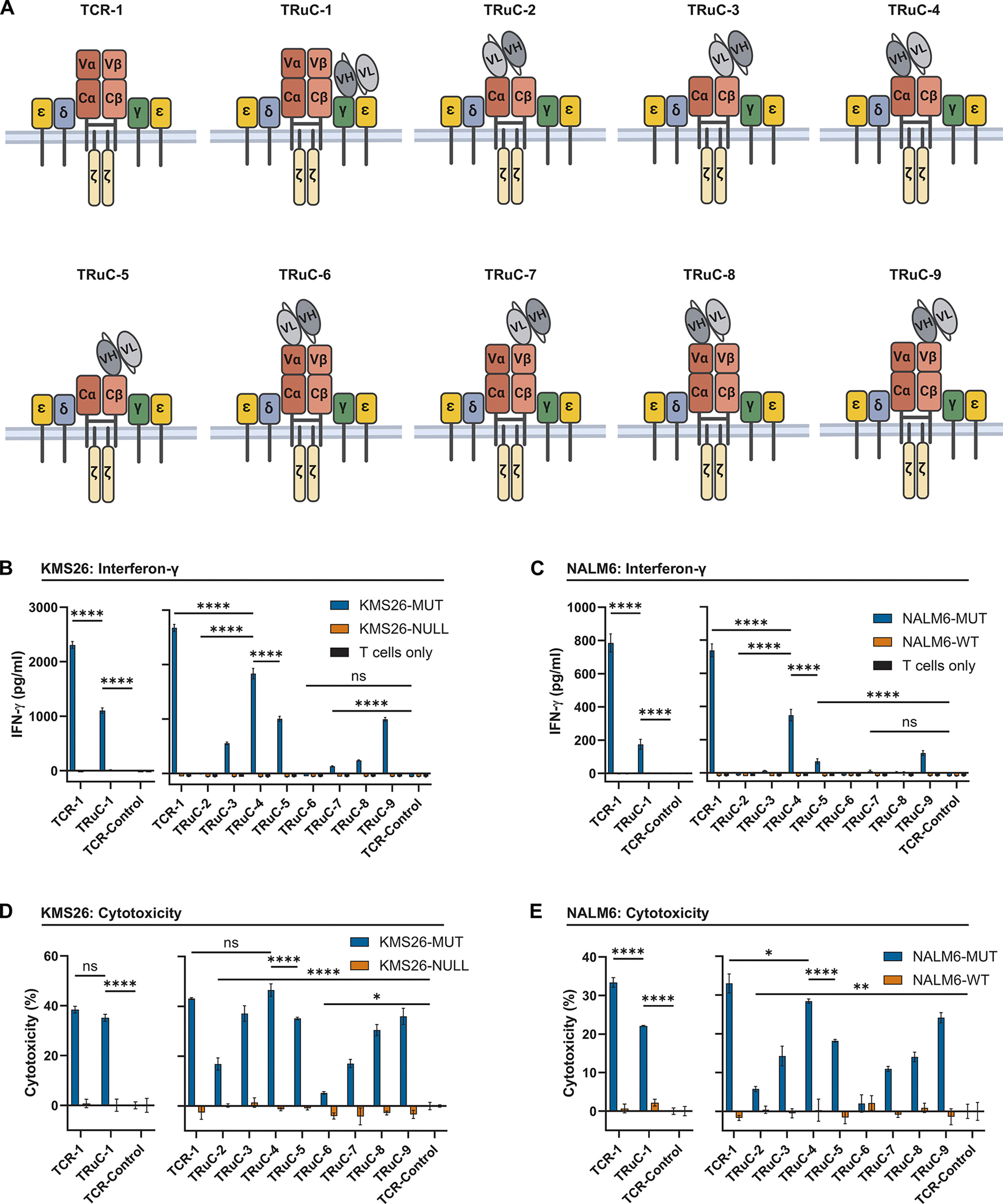
Hybrid TRuCs can signal at endogenous p53RH antigen densities. (A) Diagrams of TCR-1 and TRuC-1 through -9 demonstrate the attachment of the H2-scFv to the N-terminus of CD3γ (TRuC-1), the TCRα or β constant domains (Cα, Cβ) (TRuC-2 to 5), or the TCRα or β variable domains (Vα, Vβ) of the full length TCR (TRuC-6 to 9). The H2-scFv was attached in the VLVH orientation (TRuC-1, 4, 5, 8, 9) or VHVL orientation (TRuC-2, 3, 6, 7). TRuC-1 was introduced into the *CD3G* locus, whereas all other constructs were introduced into the *TRAC* locus. For TCR-1, human or modified murine constant domains were used and found to be equivalent (fig. S19). Human constant chains are used on the left subpanels of subsequent plots whereas modified murine constant domains are used on the right subpanels. (B) Modified T cells (1 × 10^4^) were incubated with the KMS26 isogenic cell set (5 × 10^4^) for 20 hrs. Conditioned supernatant was analyzed for IFN-γ by ELISA. (C) Modified T cells (1 × 10^4^) were incubated with the NALM6 isogenic cell set exactly as in B. (D) Modified T cells (1 × 10^4^) were incubated with the KMS26 isogenic cell set (5 × 10^4^) for 20 hrs followed by a bioluminescence assay to quantify cytotoxicity (E) Co-culture with NALM6 isogenic cell set exactly as in D. Data are shown as means ± SD of three technical replicates for all conditions except T Cells Only, which represent two technical replicates. Comparisons between TCR-1, TRuC-1, TRuC-4, and TRuC-5 are representative of N = 2 independent experiments. ****P < 0.0001, *P < 0.05, ns (not significant) by two-way ANOVA with Tukey’s multiple comparison test.

Neither antigen binding capacity as measured by flow cytometry (fig. S1B) nor reactivity at higher antigen densities (Fig. 1B) could explain the differences in reactivity of the TCR and CAR against endogenous pHLA densities (Fig. 1, C to F). Another explanation for the differences in reactivity might be in the downstream signaling that is different in TCR-1 compared to CAR-1 T cells (Fig. 1A), as has been previously proposed (44, 49, 50). In TCR-1 cells, the signaling complex includes multiple subunits encoded by distinct genes, whereas in CAR-1 T cells, the signaling comes only from the intracellular domain of the CAR protein. To evaluate whether incorporation of a MANAbody within the TCR complex could increase sensitivity to low antigen densities, we appended the scFv of the H2-CARs to the N-terminus of the CD3γ subunit of the TCR complex and used CRISPR HDR to replace the endogenous *CD3G* gene in primary human T cells, creating T-cell receptor fusion construct-1 (TRuC-1, Fig. 2A). Similarly, we appended the scFv to the N-terminus of the TCRα or TCRβ subunits in various ways and introduced the new construct to the *TRAC* locus, abrogating the expression of the endogenous *TRAC* gene, creating eight different T-cell types named TRuC-2 to TRuC-9 (Fig. 2A). In these constructs, the *TRBC* gene was also disrupted with CRISPR. Although a subset of these constructs have the same architecture as previously described TRuC cells, we used CRISPR-HDR to achieve site specific integration and to eliminate competition from the endogenous TCR chains rather than the lentiviral overexpression originally used with TRuCs (51). These constructs will be referred to as Tier 2 constructs.

All Tier 2 TRuC constructs bound the p53RH antigen when assessed by flow cytometry (fig. S3). Seven of the nine tested types of Tier 2 TRuC T cells could be activated to various extents as assessed by IFN-γ secretion after co-culture with cancer cells expressing low densities of the p53RH antigen (*P* < 0.0001; Fig. 2, B and C), unlike CAR T cells (Fig. 1, C and D). Subtle differences in Tier 2 TRuC structures had a major impact on reactivity and function. For example, orientation of the variable heavy (VH) and variable light chains (VL) of the H2-scFv with respect to the N-terminus of the receptor (VHVL or VLVH) was critical for IFN-γ production (*P* < 0.0001 comparing TRuC-2 with TRuC-4 in Fig. 2, B and C). Many of the Tier 2 TRuC T cells were also able to kill target cells bearing low densities of p53RH antigen as shown by detectable cytotoxicity (*P* < 0.05, Fig. 2, D and E). Cytotoxicity was observed with some Tier 2 TRuC T cell types even when they did not secrete IFN-γ (*P* < 0.05 for TRuC-2). Non-concordance between cytokine secretion and cytotoxicity has been previously described in other studies on T cells (48, 60–62). The specificity of all Tier 2 TRuC T cells was documented by performing identical assays with KMS26 or NALM6 cells devoid of the p53RH antigen (Fig. 2, B to E).

### STAR T cells match conventional TCR T cell antigen sensitivity

**Fig. 3.**
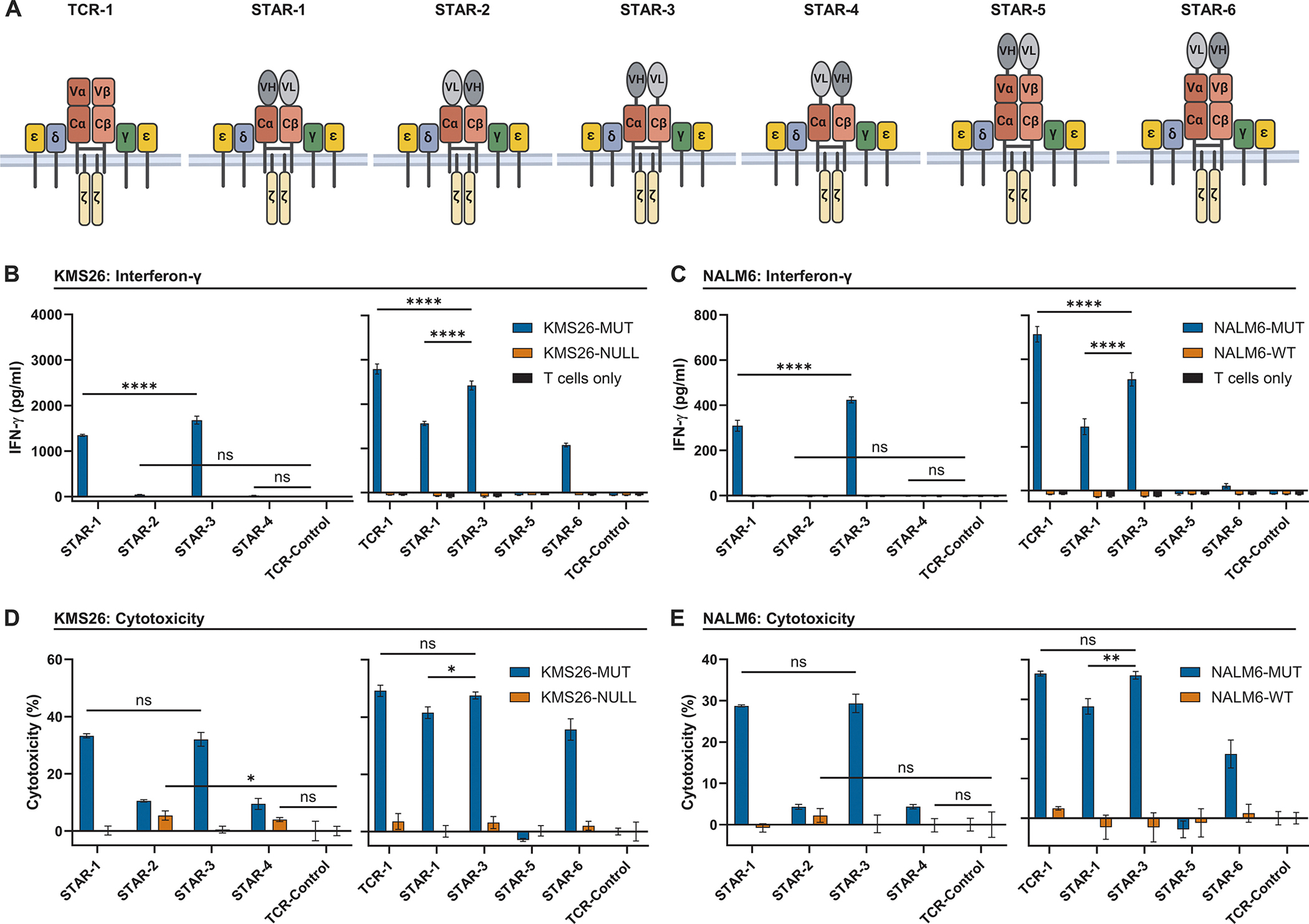
STARs have comparable potency to conventional TCRs. (A) Diagrams of TCR-1 and STAR-1 through -6 depict the attachment of the split H2-scFv VL and VH domains to the TCRα or β constant domains (Cα, Cβ) without a linker (STAR-1, -2) or with a 5 amino acid “EAAAK” linker (STAR-3, -4). STAR-5 and -6 show attachment of the split VL and VH domains to the N-termini of the TCRα and β variable domains (Vα, Vβ) of the full length TCR through a 5 amino acid “GGGGS” (G4S) linker. All depicted constructs utilize modified murine constant domains and human variable domains. (B) Modified T cells (1 × 10^4^) were cultured with KMS26 isogenic cell set (5 × 10^4^) for 20–21 hrs. Conditioned supernatant was analyzed for IFN-γ by ELISA. (C) Co-culture conditions exactly matched B except the NALM6 isogenic cell set was used. (D) Modified T cells (1 × 10^4^) were cultured with KMS26 isogenic cell set (5 × 10^4^) for 20–21 hrs. A bioluminescence assay was used to quantify cytotoxicity. (E) Co-culture conditions exactly matched D except the NALM6 isogenic cell set was used. Data are shown as means ± SD of three technical replicates, for all conditions except T Cells Only, which represent two technical replicates. Comparisons of TCR-1, STAR-1 and STAR-3 are representative of N = 4 independent experiments and n = 2 healthy donors. ****P < 0.0001, **P < 0.01, *P < 0.05. ns, not significant, by two-way ANOVA with Tukey’s multiple comparison test.

Although the Tier 2 TRuC T cells could react with target cells bearing low antigen densities, their reactivity was inferior to a conventional TCR (*P* < 0.0001, Fig. 2, B to E). In an effort to further emulate naturally occurring TCRs in a manner similar to previously described synthetic TCR and antigen receptor (STAR) or HLA-independent TCR (HIT) designs, we split the VH and VL domains of the H2-scFv and used them to replace the normal recognition domains of the TCRα and β chains to create Tier 3 T cells (STAR-1 and -2, Fig. 3A) (52, 53). We also separated the VH and VL domains from the Cα and Cβ domains by insertion of a rigid five amino acid “EAAAK” linker (STAR-3 and -4, Fig. 3A), which we hypothesized might allow for improved approximation of the H2 variable domains and maintenance of structural rigidity (63). Finally, we appended the individual VH and VL domains to the N-termini of the Vα and Vβ domains of the intact TCR through a flexible five amino acid “GGGGS” (G4S) linker (STAR-5 and -6, Fig. 3A). A more flexible G4S linker was chosen to accommodate the wider distance between Vα and Vβ N-termini compared to the Cα and Cβ N-termini of the TCR. In contrast with the lentiviral-overexpression used in the original STAR approach, CRISPR-based engineering of primary T cells eliminated competition from the endogenous TCR chains, generating truly monospecific T cells (52). Although the HIT approach utilized CRISPR to insert the HLA-independent receptor under control of the endogenous *TRAC* promoter, we and others have found that EF1α-driven rather than endogenous *TRAC*-driven expression of transgenic constructs integrated within the *TRAC* locus produces greater functionality of T cells against low density pHLAs (fig. S4).

After CRISPR-engineered integration into primary human T cells, all Tier 3 STARs bound the p53RH antigen, as measured by flow cytometry (fig. S5). Tier 3 STAR T cells were then co-cultured with KMS26-MUT or NALM6-MUT target cells and evaluated for cytokine secretion (Fig. 3, B and C) and cytotoxicity (Fig. 3, D and E) as described above. There was no reactivity as shown by IFN-γ secretion when the Vα domain of the TCR was replaced by VL of the H2-scFv and Vβ was replaced by VH with or without the linker (*P* > 0.05 for STAR-2 and STAR-4; Fig. 3, B and C). These replacements in STAR-2 also resulted in T cells that reacted less specifically with their target antigens, as assessed by cytotoxicity against KMS26-NULL isogenic cells without the mutant antigen (*P* < 0.05; Fig. 3D). Overall, the STAR receptor design with the optimum performance was STAR-3, in which the TCR Vα and Vβ were substituted with the H2-scFv VH and VL chains, respectively, with the addition of the rigid EAAAK linker (Fig. 3A). Cytotoxicity of the STAR-3 T cells was not significantly different than that of T cells containing their natural receptors, TCR-1 (*P* > 0.05; Fig. 3, D and E). IFN-γ secretion for STAR-3 T cells was lower than TCR-1 T cells but was greater than all other STAR T cell variants (*P* < 0.0001; Fig. 3, B and C).

To further substantiate the equivalence of the STAR-3 T cells with T cells expressing naturally occurring TCRs, we evaluated three TCRs directed against the p53RH antigen in addition to the TCR already tested in patients with cancer (TCR-1). The TCRs had affinities ranging from 1.1 to 39.9 μM (56, 64), and each was inserted into the *TRAC* locus of primary T cells using CRISPR-based technologies (fig. S1A) to create TCR-2, -3, and -4. STAR-3 T cells elicited greater IFN-y secretion than three of four TCRs after co-culture with KMS26-MUT cells (*P* < 0.0001) and the same amount of IFN-y secretion as one TCR after co-culture with NALM6-MUT cells (*P* > 0.05; fig S6B). STAR-3 T-cell cytotoxicity was greater than (*P* < 0.0001) or equivalent to (*P* > 0.05) the cytotoxicity of all four naturally occurring TCRs after co-culture with both cancer cell types expressing the p53RH antigen at low densities (fig. S6, C and D).

**Fig. 4.**
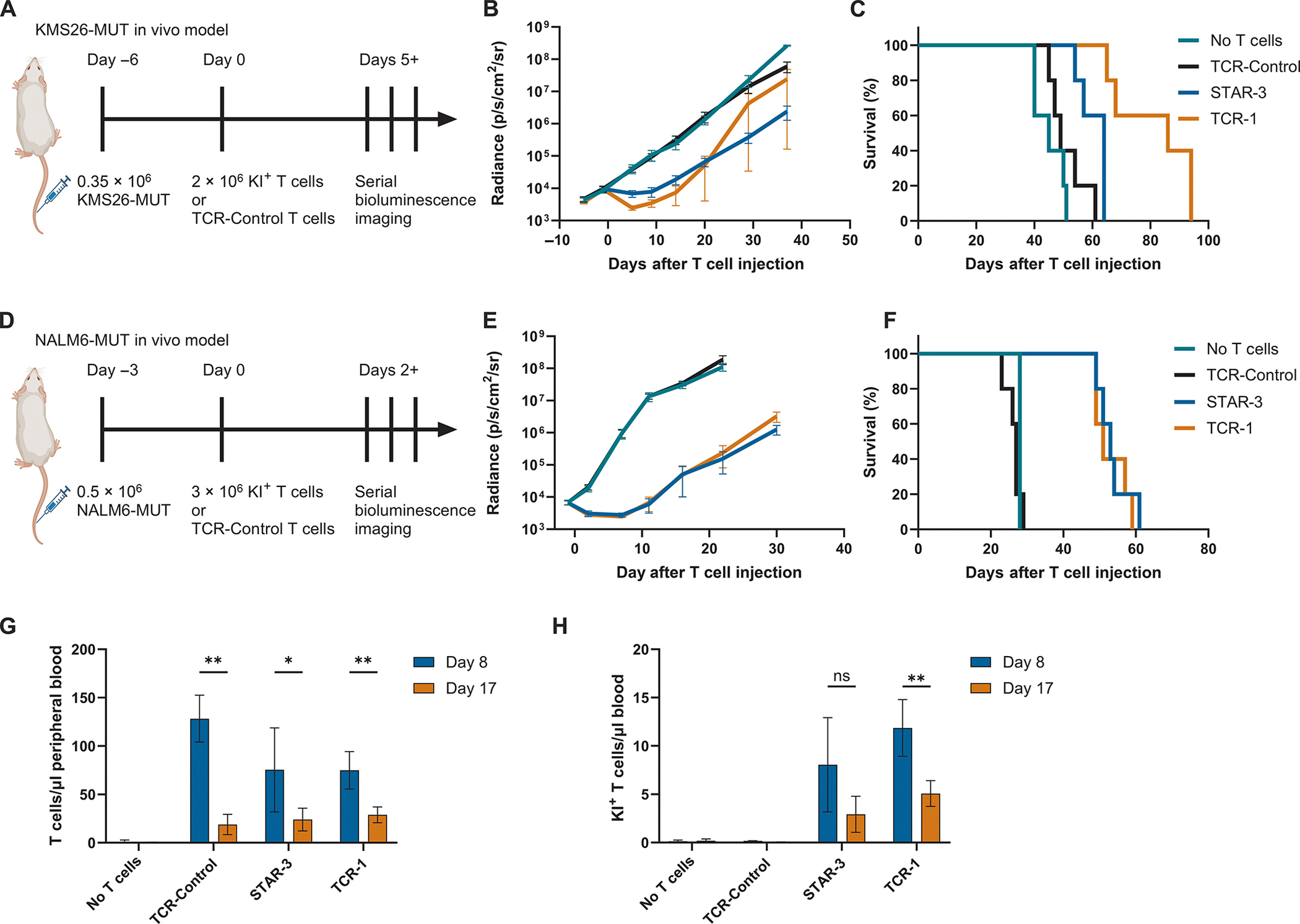
STAR and TCR demonstrate in vivo activity. (A) Schematic of KMS26-MUT in vivo model timeline. NSG mice were injected through the tail vein with 0.35 × 10^6^ KMS26-MUT cells on day -6. Mice were randomized based on bioluminescence imaging (BLI) signal on day -1. Either 2 × 10^6^ knock-in (KI+) T cells normalized to 18% KI frequency with TCR-Control T cells or 11.1 × 10^6^ TCR-Control T cells were injected through the tail vein on day 0. (B) BLI measurements of KMS26-MUT tumor cells in treated mice. Data represent means ± SEM, *n* = 5. BLI measurements were discontinued either when mice died or when treatment conditions showed consistent cancer cell growth over serial measurements spanning more than two weeks. Two-way ANOVA with Holm-Šídák multiple comparison correction was used to compare treatment groups with at least 4 surviving mice (*P* < 0.0001 comparing TCR-Control to STAR-3, *P* < 0.001 comparing TCR-Control to TCR-1). (C) Kaplan-Meier survival curves for mice in the KMS26-MUT in vivo model. Log-rank Mantel–Cox test with Bonferroni correction was used (*P* < 0.05 comparing STAR-3 and TCR-1 as well as TCR-Control to STAR-3; *P* > 0.05 comparing STAR-3 to TCR-Control). (D) Schematic of NALM6-MUT in vivo model timeline. NSG mice were injected through the tail vein with 0.5 × 10^6^ NALM6-MUT cells on day -3. Mice were randomized based on BLI signal on day -1. Either 3 × 10^6^ KI+ T cells normalized to 16% KI+ frequency with TCR-Control T cells or 18.5 × 10^6^ TCR-Control T cells were injected through the tail vein on day 0. (E) BLI measurements of NALM6-MUT tumor cells in treated mice. Data represent means ± SEM, *n* = 5. Curves for No T cells and TCR-Control are truncated because all mice died before the day 30 timepoint. BLI measurements were discontinued when treatment conditions showed consistent cancer cell growth over serial measurements spanning more than two weeks. Two-way ANOVA with Holm-Šídák multiple comparison correction was used to compare treatment groups with at least 4 surviving mice (*P* < 0.0001 comparing TCR-Control to STAR-3 and TCR-1). (F) Kaplan-Meier survival curves for mice in the NALM6-MUT in vivo model. Log-rank Mantel–Cox test with Bonferroni correction was used (*P* < 0.05 comparing TCR-Control to STAR-3 and TCR-1; *P* > 0.05 comparing STAR-3 to TCR-1). (G) Numbers of total T cells in the peripheral blood of mice were quantified by flow cytometry on days 8 and 17 after T-cell injection. (H) Numbers of knock-in (KI+) T cells were quantified on days 8 and 17. Data represent mean ± SD of measurements from five mice, n = 5. **P < 0.01, *P < 0.05, ns (not significant) by paired t-tests with Holm-Šídák multiple comparison correction for G and H. Experiments in this figure were performed N = 1 time.

To determine whether the improved function of STAR-3 T cells in vitro could also be observed in vivo, KMS26-MUT cells were intravenously injected into NOD.Cg-Prkdc^scid^Il2rg^tm1Wjl^/SzJ (NSG) mice. Following documentation of tumor engraftment six days after implantation, the mice were treated with a single dose of STAR-3, TCR-1, TCR-Control T cells, or no T cells (Fig. 4A). Delayed growth of the established tumors was observed after treatment with either STAR-3 (*P* < 0.0001) or TCR-1 cells (*P* < 0.001; Fig. 4B). However, all tumors eventually relapsed, with mice dying of their cancers between 1.5 and 3 months after the initiation of T-cell therapy (Fig. 4C). To validate these results in a different in vivo model, a similar experiment was conducted with NALM6-MUT cancer cells instead of KMS26-MUT cells (Fig. 4D). Equivalent delays in tumor growth were observed after treatment with STAR-3 or TCR-1 cells (*P* < 0.0001; Fig. 4E). However, as in the KMS26-MUT model, the NALM6-MUT cancers soon relapsed, and the mice died of their cancers 2 months after the initiation of T-cell therapy (Fig 4F). On days 8 and 17 after T cell injection in the NALM6-MUT model, peripheral blood was analyzed for the persistence of the engineered T cells. Neither TCR-1 nor STAR-3 T cells had expanded at the Day 17 time point compared to Day 8: TCR-1 T cell numbers had contracted (*P* < 0.01) and STAR-3 T cell numbers were stable (*P* > 0.05; Fig. 4, G and H) even though the tumors were continuing to expand (Fig. 4E). This result suggested that the tumors recurred in these model systems because the T-cell expansion was not sustained. Such un-sustained T-cell expansion associated with tumor relapse has been noted in previous studies in mice and humans, including prior studies on TCR-like CARs such as STAR and HIT (11, 13, 18, 52, 53, 65).

### Co-stimulation boosts activity of conventional TCRs and STARs

Considering the transient in vivo tumor control and un-sustained T-cell proliferation obtained with TCR-1 or STAR-3 T cells, we sought to identify additional co-stimulatory components that might augment and prolong T-cell activity against cancer cells expressing low densities of surface antigen. Approaches to achieve such goals have been described previously (18, 53, 66–68). We sought to expand on these approaches previously tested by implementing co-stimulation strategies that were independent of tumor-derived signals, other than the targeted pHLA antigenic signal itself. We therefore incorporated constitutively active signaling proteins into the T cells or integrated co-stimulatory domains directly into components of the TCR complex. We also explored the incorporation of co-signaling proteins that would be responsive to signals derived from the T cells themselves after initial activation, such as Fas ligand (67).

We began this exploration using TCR-1 T cells rather than STAR-3 T cells, and expressed constitutively active STAT3, STAT5, or interleukin-7 receptor alpha (IL7Rα) variants (69–71). We also expressed Fas extracellular and transmembrane domains linked to 4-1BB, OX40, IL7Rα, or MyD88-CD40 (MC) signaling domains (67, 72). Finally, we linked the MC signaling domains to the transmembrane domain of the TCRβ chain. In each case, the co-stimulatory modules were introduced into the *TRAC* locus through CRISPR-based technologies along with TCR-1 (fig. S7A), generating Co-TCR (Co-Stimulatory TCR) T cells of nine types (fig. S7B). These constructs are termed Tier 4 constructs. All Tier 4 Co-TCR constructs were expressed in T cells as measured by flow cytometric staining with p53RH tetramer (fig. S7C). When functionally tested in co-culture with NALM6-MUT cells expressing p53RH antigen at low densities, two of the Co-TCR cell types stood out: those with MyD88-CD40 stimulating domains (Co-TCR-1 and -2). Co-TCR-1 and -2 T cells secreted higher concentrations of IFN-γ (*P* < 0.0001), proliferated to a greater extent (*P* < 0.0001), and expressed fewer co-inhibitory receptors than the other seven types (*P* < 0.0001; fig. S8, A, B, and C). Co-TCR-1 and Co-TCR-2 T cells also displayed improved cytotoxicity compared to TCR-1 T cells after seven stimulations with NALM6-MUT cells (*P* < 0.01; fig. S8, D and E). The specificity of all Co-TCR T cell types was documented by the absence of cytokine secretion and cytotoxicity after co-culture with isogenic cancer cells devoid of the p53RH antigen (*P* > 0.05; fig. S8, F and G).

**Fig. 5.**
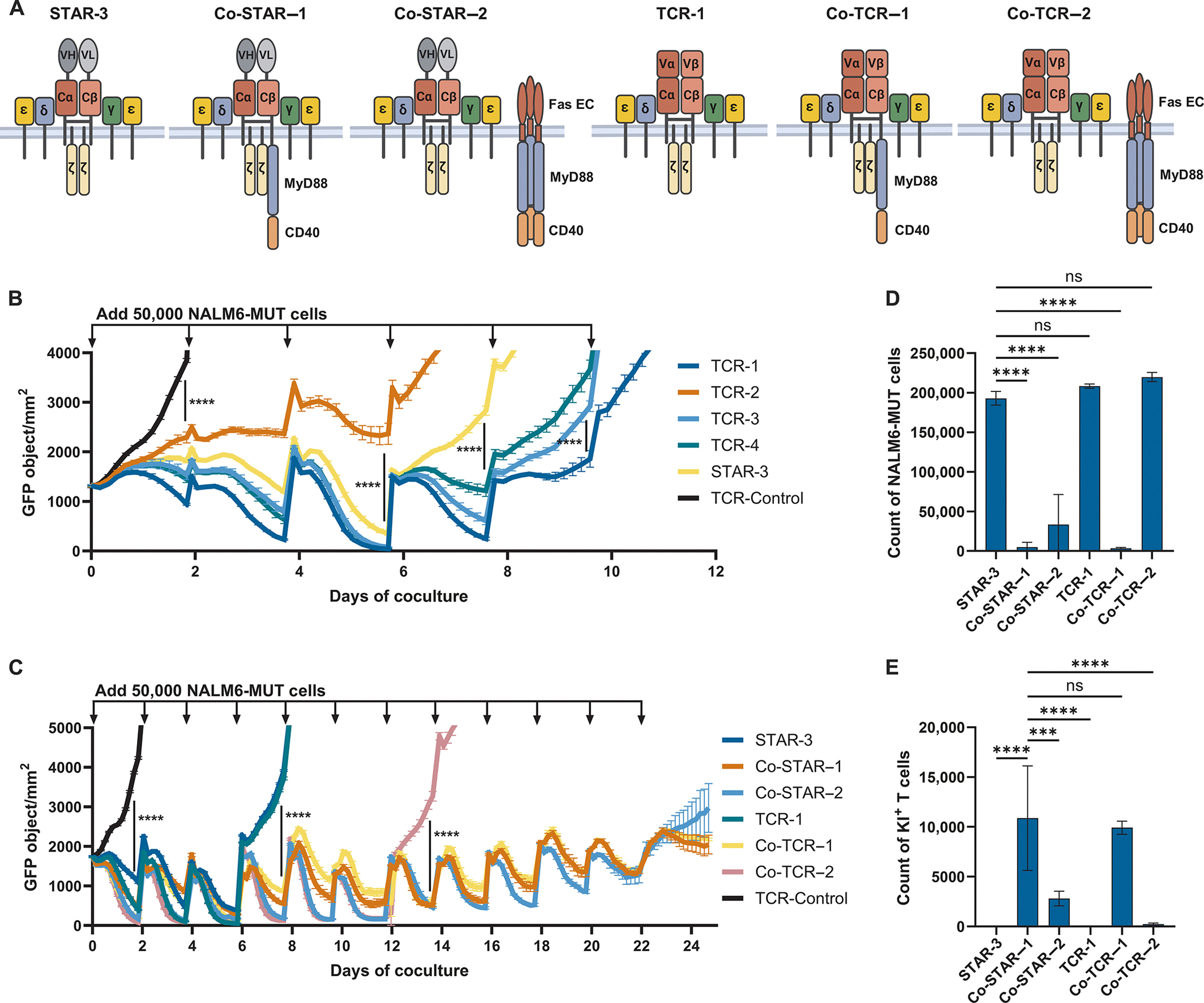
Co-stimulation improves long-term in vitro function of STAR and TCR. (A) Schematic depicting co-stimulation modified STAR-3 and TCR-1. MyD88 and CD40 (MC) domains linked to the transmembrane domain of the TCRβ chain generate Co-STAR-1 and Co-TCR-1, whereas MC domains linked to the transmembrane domain of Fas generate Co-STAR-2 and Co-TCR-2. (B) STAR-3 was compared to four patient-derived TCRs. Modified T cells (1 × 10^4^) were cultured with NALM6-MUT cells expressing GFP (5 × 10^4^) in the absence of exogenous cytokines. Every 48 hrs, 5 × 10^4^ NALM6-MUT cells were added to the co-culture. Live cell imaging was used to quantify cancer cells. (C) STAR-3 was compared to constructs containing MC domains. The experiment was set up exactly as described in B. Data are representative of means ± SEM of three technical replicates (B) or four technical replicates (C). (D) Flow cytometric quantification of NALM6-MUT cells on day 25 of the multiple stimulation assay shown in C. (E) Flow cytometric quantification of knock-in positive (KI+) T cells on day 25 of the multiple stimulation assay shown in C. Data are shown as means ± SD of four technical replicates. All data are representative of N = 2 independent experiments and n = 2 healthy donors. ****P < 0.0001, ***P < 0.001, ns (not significant) by two-way ANOVA (B and C) or one-way ANOVA (D and E) with Tukey’s multiple comparison test.

Based on these results with TCR-1 T cells, we analogously engineered the most potent Tier 3 STAR T cells (STAR-3, Figure 3) to express MC domains (Fig. 5A), generating additional Tier 4 constructs. The resulting T cells were named Co-STAR-1 and Co-STAR-2 (Co-stimulatory STAR). After co-culture with KMS26-MUT or NALM6-MUT target cells, the MC domains in Co-STARs showed enhanced IFN-γ production over that observed in the unmodified STAR-3 or TCR-1 T cells (*P* < 0.0001; fig. S8, H and I). The specificity of Co-STAR cells was retained, as shown by a lack of IFN-γ production after co-culture with KMS26 or NALM6 cells devoid of the p53RH antigen (*P* > 0.05; fig. S8, H and I).

### Long-term tumor control is enabled by co-stimulation in Co-STAR T cells

The purpose of including co-stimulatory domains in Co-STAR and Co-TCR T cells was to prolong and enhance the ability of T cells to control cancer cell growth. To better mimic the repeated antigen exposure that occurs in tumors of patients, we used multi-stimulation assays (MSAs) in which new GFP-labeled cancer cells (but not new T cells) were added to the co-cultures every other day and growth was monitored over relatively long periods of time (73, 74). STAR-3 T cells, as well as TCR-1, -2, -3, and -4 T cells, were all initially able to slow the growth of NALM6-MUT cancer cells (*P* < 0.0001; Fig. 5B). However, the T cells failed to control growth during re-challenge after six to eight days in culture. This result was expected given the failure of the same T cells to mediate long-term control of tumors in mice (Fig. 4, A to F). However, with the addition of MC co-stimulatory domains to the T cells, cancer cell growth could be controlled much longer than non-co-stimulated STAR-3 and TCR-1 T cells (*P* < 0.0001), with failure evident only after three weeks of co-culture for three of the four T-cell types tested: Co-STAR-1, Co-STAR-2, and Co-TCR-1 T cells (Fig. 5C). Flow cytometric quantification of viable NALM6-MUT cells at the end of the MSA confirmed the superior cytotoxicity of Co-STAR-1, Co-STAR-2, and Co-TCR-1 T cells (*P* < 0.0001; Fig. 5D). This prolonged control was mirrored by the persistence of the T cells (Fig. 5E). T cells with MC co-stimulatory domains attached to the transmembrane domain of TCRβ (Co-STAR-1 and Co-TCR-1) were present at greater numbers at the end of the MSA than all other conditions (*P* < 0.001), including T cells in which MC co-stimulatory domains were attached to the transmembrane domains of Fas (Co-STAR-2 and Co-TCR-2; Fig. 5E).

**Fig. 6.**
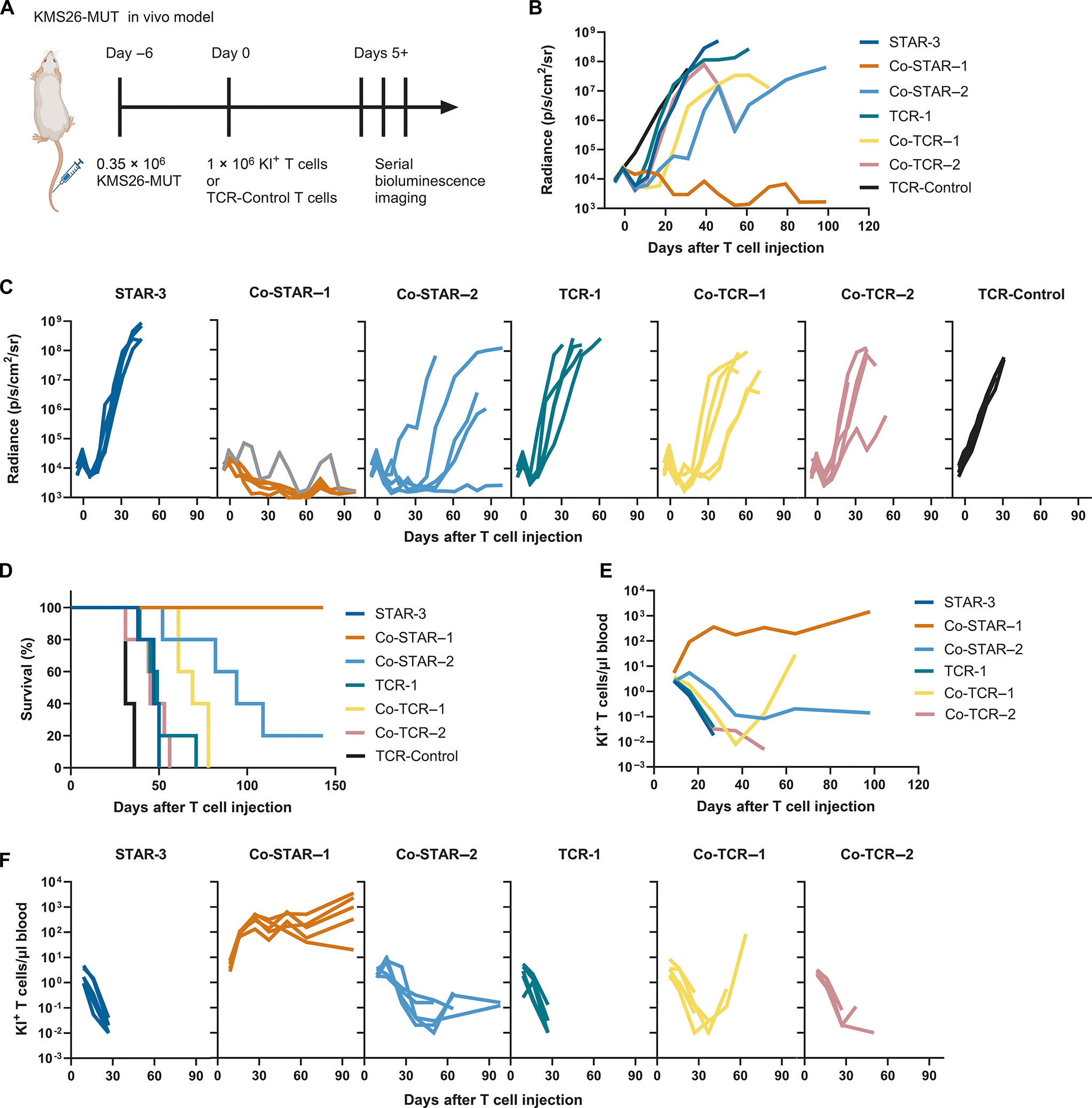
Co-STARs demonstrate prolonged activity in vivo. (A) Schematic showing the design of the in vivo experiment. NSG mice (*n*=5 mice per treatment group) were inoculated through the tail vein with 0.35×10^6^ KMS26-MUT cells on day -6. Mice were randomized based on BLI signal on day -1. Tail vein injection of modified T cells (either 1 × 10^6^ knock-in+ (KI+) T cells normalized to 10% KI frequency with TCR-Control T cells or 1 × 10^7^ TCR-Control T Cells) was performed on day 0. Approximately weekly BLI imaging was used to track cancer cell growth. (B) Radiance measurements for treatment groups are displayed as means. Curves are truncated when all mice in a treatment arm died before the specified timepoint. BLI measurements were discontinued 99 days after initial treatment. Two-way ANOVA with Holm-Šídák multiple comparison correction was used to compare all treatment groups with at least 4 surviving mice through day 24 (*P* < 0.01 comparing TCR-Control to Co-STAR-1, Co-STAR-2, and Co-TCR-1; *P* > 0.05 comparing TCR-Control to STAR-3, TCR-1, and Co-TCR-2), through day 39 (*P* < 0.01 comparing Co-STAR-2 and Co-TCR-2), though day 54 (*P* < 0.01 comparing Co-STAR-1 and Co-TCR-1), and through day 86 (*P* < 0.01 comparing Co-STAR-1 and Co-STAR-2; B). (C) The same radiance data in B are displayed for individual mice by treatment group. One curve is gray in Co-STAR-1 to indicate that multiple tumor outgrowths occurred in the same mouse. (D) Kaplan-Meier survival curves of seven treatment groups, *n*=5 mice per group. Log-rank Mantel-Cox test with Bonferroni-Holm correction was used (*P* < 0.05 comparing Co-STAR-1 to TCR-1, Co-TCR-1, and Co-TCR-2; *P* > 0.05 comparing Co-STAR-1 and Co-STAR-2). (E) Quantification of KI+ T cells in peripheral blood of mice using flow cytometry. Measurements are shown as means, n = 5 mice per treatment group. The curves are truncated either when all mice within a treatment group died or when the number of KI+ T cells detected in peripheral blood by flow cytometry was zero. Two-way ANOVA with Holm-Šídák multiple comparison correction was used to compare all treatment groups through day 27 (*P* < 0.0001 comparing Co-STAR-1 to all other groups). (F) The same KI+ T cell concentrations from E are displayed for individual mice by treatment group. Data in this figure are from N = 1 experiment.

To extend these observations to the in vivo setting, we returned to our KMS26-MUT mouse model (Fig. 6A). By day 24 after intravenous T-cell injection, non-co-stimulated Tier 3 T cells (TCR-1 and STAR-3) and Co-TCR-2 T cells were unable to control KMS26-MUT tumors better than TCR-control T cells (*P* > 0.05), whereas Co-STAR-1, Co-STAR-2, and Co-TCR-1 T cells demonstrated anti-tumor activity (*P* < 0.01; by two-way ANOVA through day 24; Fig. 6, B and C). Co-STAR T cells were more effective than Co-TCR T cells with the same MC domains as shown by comparing tumor growth kinetics of Co-STAR-1 with Co-TCR-1 (*P* < 0.01) and Co-STAR-2 with Co-TCR-2 (*P* < 0.01; Fig. 6, B and C). The MC domain showed greater long-term control of tumor growth when attached to TCRβ (Co-STAR-1) than when attached to Fas (Co-STAR-2; *P* < 0.01; Fig. 6, B and C). Mice treated with Co-STAR-1 T cells survived longer than mice treated with T cells modified with any type of natural or co-stimulated TCR (TCR-1, Co-TCR-1, and Co-TCR-2), with all treated mice alive at 5 months (*P* < 0.05; Fig. 6D).

A plausible explanation for the superiority of Co-STAR-1 cells over the other T-cell types was discovered by monitoring the number of modified human T cells in the mice during the course of this experiment. The degree of expansion and persistence of Co-STAR-1 cells was greater than those of any other type of T cells evaluated (*P* < 0.0001; Fig. 6, E and F). The difference in expansion of T cells in vivo between Co-STAR-1 and the other types of T cells was two to three orders of magnitude. Further evidence of the importance of the prolonged expansion of Co-STAR-1 T cells in vivo was supported by the data in Fig. 6C: there were two minor relapses of tumor at 39 days and at 71 days following therapy in the same mouse (indicated by the gray curve). Both relapses were transient, presumably due to the continued presence and functionality of Co-STAR-1 T cells in that mouse (Fig. 6, E and F).

We also evaluated the ability of the engineered T cells to control the growth of a second tumor model in vivo (fig. S9A). Overall, the relative performance of the various T-cell types was similar in mice bearing NALM6-MUT tumor cells compared to KMS26-MUT tumor cells. For example, Co-STAR T cells were more potent than their corresponding Co-TCR T cells modified with the same MC domains in terms of tumor growth kinetics (P < 0.01; Fig. S9, B and C). Co-STAR-1 T cells again mediated longer survival than T cells equipped with natural or co-stimulated TCRs (TCR-1, Co-TCR-1, and Co-TCR-2; *P* < 0.05; fig. S9D). The degree of expansion and persistence of Co-STAR-1 cells was also greater than any other type of T cells evaluated (*P* < 0.01; fig. S9, E and F). The specificity of Co-STAR-1 T cells was documented by experiments in mice bearing isogenic NALM6-WT tumor cells (harboring the normal arginine rather than the mutant histidine at amino acid 175 of p53) (fig. S10A). Both the growth of NALM6-WT cells and mouse survival were unaffected by Co-STAR-1 or any other of the engineered T cells assessed (*P* > 0.05; fig. S10, B and C). Moreover, the expansion of Co-STAR-1 cells, but not the other T cell types, in mice occurred even in the absence of p53RH stimulation in vivo (*P* < 0.0001; fig. S10D). No toxicity, as assessed by weight loss relative to the initial timepoint, was observed in mice treated with Co-STAR-1 T cells in both the NALM6-WT model (*P* > 0.05 indicating stable weights; Fig. S10E) and the NALM6-MUT model (*P* < 0.05 indicating increased weights; Fig. S10F).

However, there was one major difference in the therapeutic efficacy between the KMS26-MUT and NALM6-MUT models. In the KMS26-MUT model, Co-STAR-1 treatment induced complete remissions in every mouse, and the remissions lasted throughout the entire duration of the experiment (survival of 5 months, Fig. 6D). By contrast, in the NALM6-MUT model, mice began to succumb to their tumors two months following Co-STAR-1 treatment, with four of the five treated mice dead by day 71 (fig. S9D). Based on prior studies with T-cell therapeutics in mice and humans, T-cell treatment failures are typically due to a lack of persistence of the T cells or loss of antigen on the target cells. We could exclude the first explanation because Co-STAR-1 T cells persisted in the mice even after their NALM6-MUT tumors began to grow (compare fig. S9C to S9F). The second explanation – loss of antigen on the target cells – appeared to be the correct one. Flow cytometry revealed that all four of the Co-STAR-1 treated mice that eventually died from their cancer had lost the HLA-A*02:01 component of the p53RH antigen on their circulating cancer cells (fig. S11). By contrast, in most mice treated with either Co-STAR-2 or Co-TCR T cells, most of which succumbed to their tumors earlier than those treated with Co-STAR-1 T cells, the circulating cancer cells retained the expression of HLA-A*02:01 on their surface. In these mice, tumor relapse was apparently due to the disappearance of the therapeutic T cells (fig. S9, E and F).

### Co-STAR-1 modulates cytokine secretion and tonic signaling

To examine the effects of the MC domain on Co-STAR-1 function, transcriptomic changes in the CD4+ and CD8+ subsets of unedited, STAR-3, and Co-STAR-1 T cells were examined after an 18-hour co-culture with KMS26-MUT, KMS26-NULL, or no target cells. In general, hundreds of genes were found to be differentially expressed in every comparison made (table S2). The gene encoding IL-2 (*IL2*) was one of the most upregulated genes in Co-STAR-1 T cells compared to STAR-3 T cells after activation with KMS26-MUT cells, demonstrating a 22-fold increase in CD8+ T cells and 12-fold increase in CD4+ T cells (*p-adjusted* < 0.0001; fig. S12 and S13A). No upregulation of the gene encoding IL-2 was observed after culturing Co-STAR-1 T cells with KMS26-NULL target cells or in the absence of target cells (*p-adjusted* > 0.05; fig. S12 and S14). Concomitant upregulation in STAR-3 and Co-STAR-1 T cells of the genes encoding IL-2Rα (*IL2RA*) and STAT5A (*STAT5A*), along with constitutive expression of the genes encoding IL-2Rβ (*IL2RB*), the common gamma chain (*IL2RG*), and STAT5B (*STAT5B*) suggest an augmented IL-2 autocrine signaling loop in Co-STAR-1 T cells (fig. S12). Genes encoding additional cytokines and chemokines including IFN-γ (*IFNG*), IL-5 (*IL5*), IL-6 (*IL6*), and CCL22 (*CCL22*) were upregulated in Co-STAR-1 T cells compared to STAR-3 T cells after exposure to KMS26-MUT cells (*p-adjusted* < 0.001) and showed no upregulation after exposure to KMS26-NULL cells compared to no target cells, confirming the specificity of T-cell activation (*p-adjusted* > 0.05; fig. S12 to S14).

Stimulation with KMS26-MUT cells also upregulated the genes encoding nuclear factor kappa B (NFκB) subunit 2 (*NFKB2*) and NFκB inhibitor alpha (*NFKBIA*) in Co-STAR-1 T cells compared to STAR-3 T cells, both of which are upregulated by NFκB signaling as part of an auto-regulatory feedback loop (*p-adjusted* < 0.0001; fig. S12) (75). Network analysis of differentially upregulated genes in Co-STAR-1 T cells after KMS26-MUT stimulation confirmed the upregulation of multiple pathways in CD8+ Co-STAR-1 T cells that signal through NFκB, including toll-like receptor (TLR)- and MyD88-related pathways (fig. S15, A and B).

Co-STAR-1 T cells also showed gene expression changes associated with reduced apoptosis and exhaustion after co-culture with KMS26-MUT cells as demonstrated by the increased expression of the gene *SERPINB9* encoding proteinase inhibitor-9, a cytoplasmic protein that protects T cells from granzyme B-mediated activation-induced cell death, decreased expression of the gene *GZMB* encoding granzyme B in CD4+ T cells, and decreased expression of the gene *HAVCR2* encoding the co-inhibitory receptor TIM3 (*p-adjusted* < 0.0001; fig. S12). Additionally, the gene encoding cellular inhibitor of apoptosis 2 (*BIRC3*), an anti-apoptotic protein and positive regulator of canonical of NFκB signaling, was upregulated in Co-STAR-1 T cells compared to STAR-3 T cells in non-stimulating conditions (*p-adjusted* < 0.0001; fig. S12).

Tonic-signaling of synthetic receptors within T cells in the absence of cognate antigen has been shown to modulate T-cell phenotypes and anti-tumor activity (18, 65). When Co-STAR-1 and STAR-3 T cells were cultured without target cells, 127 and 258 genes were differentially expressed in CD4+ and CD8+ T cells (table S2). The gene encoding CXCL13 (*CXCL13*) was among the most differentially upregulated genes for both CD4+ and CD8+ Co-STAR-1 T cells in the absence of target cells whereas its expression was dependent on target cell activation in STAR-3 T cells (*p-adjusted* < 0.0001; fig. S12 and S13B). The concomitant expression of the gene encoding CXCR3 (*CXCR3*), a receptor for CXCL13, in CD8+ Co-STAR-1 T cells under non-stimulating conditions suggests the presence of an autonomous chemokine signaling loop (fig. S12). The gene encoding the transcription factor SOX4 (*SOX4*), which has been demonstrated to be upregulated in T cells by continuous CAR signaling, was also upregulated in Co-STAR-1 T cells in the absence of antigen (*p-adjusted* < 0.0001; fig. S12 and S13B) (76, 77). Network analysis of genes upregulated in Co-STAR-1 T cells cultured without target cancer cells identified interleukin, TLR, and non-canonical NFκB signaling pathways (fig. S15, C and D).

To confirm that the observed transcriptional differences in gene expression were also reflected translationally, Tier 4 T cells were co-cultured with either the KMS26 or NALM6 isogenic sets for a similar amount of time as the transcriptome experiment and conditioned supernatant was collected for targeted assessment of protein concentration by Luminex (figs. S16 and S17). Similar to their transcriptional profiles, IL-2, IL-5, IL-6, and CCL22 were more robustly produced by Co-STAR-1 T cells than by STAR-3 T cells after co-culture with KMS26-MUT or NALM6-MUT target cells (*P* < 0.01; fig. S16 and S17), whereas minimal to no production was detected after culture of T cells alone or co-culture with the KMS26-NULL or NALM6-WT target cells. The patterns of IL-2, IL-5, IL-6, and CCL22 production were similar between Co-STAR-1 and Co-TCR-1 T cells (fig. S16 and S17). Assessment of 10 additional markers of activation after co-culture of engineered T cells with the NALM6 isogenics confirmed the specificity of T-cell activation to the p53RH antigen (fig. S17).

We also asked whether Co-STAR or Co-TCR receptors differed in their capacity to activate CD4+ and CD8+ T cells in co-culture, as the lower affinity of the TCR-1 binding domain may render it more dependent on CD8 co-receptor engagement (56). Five days after a single stimulation with either the KMS26-MUT cells or NALM6-MUT cells, both Co-STAR and Co-TCR receptors induced expansion of both CD8+ and CD4+ T-cell subsets (*P* < 0.0001; fig. S18, A and B). The MC domains did not increase the expansion of T cells after co-culture with isogenic KMS26-NULL and NALM6-WT cells or when T cells were cultured on their own (*P* > 0.05). Inclusion of MC co-stimulation preferentially supported the expansion of CD4+ T cells, as demonstrated by the increased fraction of CD4+ T cells observed after a single stimulation with cognate antigen positive cells, with the TCRβ linkage having a more pronounced effect than Fas linkage (*P* < 0.01; fig. S18, C and D). The effect on CD4+ T-cell number was even more pronounced after a 12-day multiple stimulation assay, where the Co-STAR-1 construct preserved the highest percentage of CD4+ T cells relative to all other constructs (*P* < 0.0001; fig. S18, E and F).

## Discussion

The results described above demonstrate that it is possible to combine the advantages of TCRs and CARs, creating T cells with high-affinity, antibody-derived receptors (from the CAR component) that can specifically react with cancer cells bearing extremely low antigen densities (from the TCR component). Moreover, the inclusion of a specific co-stimulatory domain in Co-STAR T cells endowed them with the capacity to expand and remain functional in mice in an unprecedented manner, allowing long-term control of tumor growth.

Our study builds upon previous approaches for constructing hybrid TCR-like CARs and for augmenting T-cell activation with co-stimulation (50–53, 67, 69–72) while extending the functionality of synthetic receptors when exposed to cells bearing very low numbers of antigens on their cell surface. We identified a dependence of antigen sensitivity on subtle changes in receptor design, as evidenced by variable reactive capacities of Tier 2 TRuC and Tier 3 STAR T cells. The optimal design of linking the H2-scFv VL to the Cβ domain and the H2-scFv VH to the Cα domain with an “EAAAK” linker as well as the array of other STAR and TRuC variants should be a valuable resource for further studies to define the roles of light and heavy chain geometry, epitope access, proper folding, CD8 coreceptor engagement, T cell-target cell interactions, additional co-stimulation, and the transduction of mechanical force in T-cell signaling (65, 78–85).

We also demonstrated that co-stimulation provided by MyD88 and CD40 (MC domain) enabled the Co-STAR-1 and Co-STAR-2 T cells to eradicate cancer cells in mice displaying on average 1–2 pHLAs per cell, which far exceeds the lower antigen density limits previously studied in vitro or in vivo with TCR-like CARs (summarized in table S3). Linkage of the MC domain to TCRβ enabled Co-STAR-1 T cells to expand to 1,000 fold greater abundance than Co-TCR-1 T cells in mice (Fig. 6), despite these receptors only differing in the type of variable domains and their connection to the TCR constant domains. The upregulation of autocrine cytokine loops including IL-2 and CXCL13 (fig. S12 to S17), improved CD4+ T cell persistence (fig. S18), and tonic receptor signaling in Co-STAR-1 T cells (fig. S12 to S15) identify mechanisms contributing to the superior performance of Co-STAR-1 T cells (86–89). Antigen-independent tonic CAR signaling occurs through receptor oligomerization mediated by the framework regions of antibody domains, which can counterintuitively result in either impaired or improved T-cell potency depending on the context (18, 65). A similar mechanism of antibody-domain-mediated tonic signaling may explain why Co-STAR-1 T cells and not Co-TCR-1 T cells proliferated in vivo in the absence of antigen (fig. S10D) and subsequently maintained a homeostatic steady state after initial expansion in both the KMS26-MUT and NALM6-MUT in vivo models (Fig. 6E and fig. S9E). Such sustained T cell persistence may prove necessary for engineered T cells to mediate durable responses in cancer types which do not respond to conventional T-cell therapies. Future work is needed to further clarify the signaling circuits and cell states in Co-STAR-1 T cells that may contribute to antigen independent signaling and homeostatic in vivo expansion. Until these mechanisms are understood, clinical implementation may require a safety switch if (unlike in the mouse or in vitro) Co-STAR T cell proliferation becomes unregulated (90).

Co-STAR T cells appear to react to target cells containing an average of two antigenic molecules on the target cell surface. Although this is more potent than what can be achieved with other types of CAR T cells assessed in identical (Fig. 1) or similar (51–53, 70, 72) ways, two molecules per cell might be an underestimate of the actual number of molecules required for T-cell activation. The actual number of pHLA molecules on the target cell surface could have varied during the 16 hour or greater duration of the co-cultures presented here. Additionally, interferon-γ can increase the expression of HLA molecules on the surface of cancer cells in vitro or locally in vivo (91). Finally, p53RH antigen density could differ between the in vitro conditions under which the pHLA density was quantified and the in vivo conditions in which T-cell activity was benchmarked. Future technological developments, such as super resolution microscopy with picomolar affinity binding reagents, may enable quantification of the p53RH antigen and other pHLA antigens on patients’ tumors and will better define the antigen densities engineered T cells must contend with in clinical settings (43, 92, 93).

Our study has other limitations. Although long-term remissions without tumor recurrence were observed after treatment of the KMS26-MUT cancers with Co-STAR-1 T cells (Fig. 6, B to D), NALM6-MUT cancers eventually recurred following initial regressions (fig. S9). These relapses were due to the loss of the presenting HLA allele (fig. S11), a common mechanism of immune escape in cancers (56, 94–96). Overcoming such resistance mechanisms is a challenge associated with all immunotherapeutics and is the subject of intense investigation (10, 91, 97–103). A second major limitation of our study is that, although two different cancer types were evaluated with multiple different T-cell types, all T cells targeted the same antigen (p53RH), either with the same antibody chains (from the H2-scFv) or from four different TCRs. It is possible that other antigen targets or other antibodies would not be as amenable to the Co-STAR approach as was the p53RH antigen and the H2-scFv. Finally, although patterns were seen in terms of the performance of various constructs at low antigen densities, we did not delineate the precise biologic or structural mechanisms underlying these patterns. Despite the above limitations, these studies extend the realm of synthetic constructs and clearly demonstrate that synthetic constructs can approach or even exceed the activity of conventional TCRs.

In summary, our results show that Co-STAR T cells combine the advantages of CAR- and TCR-engineered T cells in a way that allows them to effectively kill cancer cells with low antigen densities in mouse models. Co-STARs address some, but certainly not all, challenges confronting T cell-based therapeutics, and seem worthy of continued investigation.

## Materials and Methods

### Study Design

The study goal was to evaluate chimeric receptor designs based on a TCR-mimic antibody fragment for the capacity to react with a low-density peptide-HLA antigen. The TCR-mimic antibody fragment used for receptor designs was the H2-scFv which targets the HMTEVVRHC peptide containing the p53 R175H mutation presented in the HLA-A*02:01 allele. Primary human T cells from healthy donors were genetically modified with CRISPR technologies and used as effector T cells. Isogenic cancer cell line pairs with and without the *TP53* R175H mutation were used to assess the potency and specificity of engineered T cells in terms of their activation and cytotoxicity. In vitro T-cell cytotoxicity was measured using bioluminescence quantification of luciferase activity, flow cytometric enumeration of specific cell populations, and live cell imaging of GFP+ target cancer cells. T-cell activation was assessed by quantification of cytokine and chemokine secretion using ELISA and Luminex assays as well as by bulk RNAseq assessment of transcriptomic changes. T-cell proliferation was quantified by flow cytometry enumeration of specific T-cell populations. For animal experiments, NOD.C*g-Prkdc*^*scid*^*Il2rg*^*tm1Wjl*^/SzJ (NSG) mice were maintained and treated in compliance with an JHU Animal Care and Use Committee–approved research protocol. Mice were intravenously injected with tumors and randomized prior to intravenous T-cell injection. No mice were excluded from the in vivo studies and experimenters were not blinded. Sample sizes for mouse models were not determined by power analysis but were determined by prior experience with mouse models. Statistical tests, number of replicates, and number of independent experiments are noted in the figure legends.

### HDRT template design and generation

Double stranded DNA HDRTs were generated by PCR amplification from plasmid templates. Plasmid templates were cloned with NEBuilder DNA Assembly HiFi (NEB, E2621L) by mixing synthesized DNA fragments (IDT gBlocks or GeneArt Strings) with a linearized pUC19 derived vector after excising the original contents with EcoRI and HindIII (Addgene, 112021). All un-annotated plasmid sequences are listed in supplementary table S5, and annotated sequences for STAR-3, Co-STAR-1, and Co-STAR-2 are included in supplementary table S6. HDRTs are designed with an EF1α promoter to drive expression of the encoded receptor and a truncated nerve growth factor receptor (tNGFR) tag. Independent protein domains are separated by furin-2A sequences. A poly-A terminator sequence is included after the stop codon. Homology arms (HAs) are approximately 300 bps in length. Unless specified in figure legends, all constructs encoding TCRα and TCRβ constant domains use murine chains modified with an additional disulfide bond and stabilizing mutations in the transmembrane of the alpha chain (52). For TCR-1, fully human TCRα and TCRβ chains without modifications were found to be functionally similar to the modified murine constant domains (fig. S19).

To produce the HDRTs, plasmid templates were PCR amplified with primers specific to the M13 forward and reverse sites using the Q5 Hot Start High-Fidelity 2X Master Mix (New England BioLabs, M0494L). For HDRTs used with a Cas9 nuclease, tCTS sites corresponding to the sgRNA sequence were employed (104). For HDRTs used with a Cpf1 nuclease, an irrelevant tCTS site was used. HDRT PCR amplicons were purified with 1x AMPure XP Reagent (Beckman Coulter Life Sciences, A63880), eluted in sterile water, and quantified with a NanoDrop Spectrophotometer (Thermo Fisher Scientific).

### CRISPR editing of primary human T cells

Peripheral blood mononuclear cells (PBMCs) were isolated by Ficoll-Paque PLUS (Cytiva, 17144002) gradient centrifugation from healthy donor leukopaks (StemCell and Charles River). CD3+ T cells were isolated by negative selection (StemCell Technologies, 17951) and activated with Dynabeads Human T-Activator CD3/CD28 (Thermo Fisher Scientific, 11132D) at a 1:1 bead-to-cell ratio in T cell media, which consisted of RPMI-1640 (ATCC, 30-2001) supplemented with 10% FBS (Cytiva, SH30070.03), 1% penicillin-streptomycin (Thermo Fisher Scientific, 15140163), 100 IU/mL recombinant human IL-2 (Proleukin, Prometheus Laboratories), and 5 ng/mL recombinant human IL-7 (BioLegend, 581908). After 48–56 hours of activation, T cells were separated from CD3/CD28 beads by two passes over a magnet and allowed to rest while CRISPR reagents were prepared. For each electroporation condition of simultaneous *TRAC* knock-in and *TRBC* knock-out, 50 pmols of each crRNA were independently mixed with 25 pmols of Alt-R A.s. Cas12a (Cpf1) Ultra (IDT, 10001273) and 37.5 pmols of Alt-R Cpf1 Electroporation Enhancer (IDT, 1076301) in a total volume of 1.27 μl of Nuclease Free Duplex Buffer (IDT, 11-01-03-01) for at least 15 minutes before combining the two RNPs at a 1:1 volume ratio. The *TRAC* and *TRBC* RNPs were then mixed with 0.5 μg HDRT diluted in 2.4 uL OptiMEM for a final volume of 5 μL per electroporation condition. Immediately prior to electroporation, T cells were centrifuged at 90g for 10 minutes, then resuspended at 0.75–1.25 × 10^6^ T cells in 20 μl P3 buffer (Lonza, V4XP-3032) and combined with the 5uL Cpf1 RNP and HDRT mixture. Cells were nucleofected in 16-well cuvettes (Lonza, V4XP-3032) with a 4D Nucleofector X-Unit (Lonza, AAF-1003X) using pulse code EH115. After nucleofection, 80 μl pre-warmed cytokine free T cell media was added to cells and the cuvette strip was placed in a 37 °C incubator for 20 min. The cells were then diluted in 1 mL of T cell media and transferred to a 24-well plate. T cell media was changed every 3 days until functional assays on days 11 or greater after initial activation.

When noted in figure legends, the CRISPR editing protocol was modified to target other genetic loci (*CD3G* instead of *TRAC* and *TRBC*) or to utilize Cas9 nuclease (IDT, 1081059) and Alt-R Cas9 Electroporation Enhancer (IDT, 1075916) instead of Cpf1 reagents. All gRNA sequences used are listed in supplementary table S4.

### Mouse xenograft models

Female NOD.C*g-Prkdc*^*scid*^*Il2rg*^*tm1Wjl*^/SzJ (NSG) mice at 6–24 weeks of age were obtained from Jackson Laboratory (005557) or the Johns Hopkins Sidney Kimmel Comprehensive Cancer Center Animal Resources facility and were maintained and treated in compliance with an JHU Animal Care and Use Committee–approved research protocol. Cancer cells and human T cells were injected individually through the tail vein in 200 μL RPMI-1640 media. The timing of injections is noted in the figure legends. Cancer cell burden was measured by quantifying bioluminescence signal. Mice were intraperitoneally injected with 150 μl RediJect d-Luciferin Ultra Bioluminescent Substrate (PerkinElmer, 770505), anesthetized with inhaled isoflurane, and then were imaged with an In Vivo Imaging System (IVIS, PerkinElmer) 6 minutes after initial substrate injection. For survival studies, mice were euthanized when hind limb paralysis was noted. For the quantification of T cells in the peripheral blood of mice, blood from the submandibular veins of mice was collected in EDTA coated microvettes (Sarstedt, NC9299309). Then, 100 μl of blood was treated with ACK lysis buffer (Quality Biological, 118-156-721) and then washed with PBS. Flow cytometry staining was conducted as specified above.

### Statistical Analysis

Mean ± standard deviation or standard error were used to summarize the data, as specified in figure legends. Statistical analyses were carried out as indicated in figure legends. Unless otherwise specified, a *P* value of < 0.05 was used to denote statistical significance. Statistical analyses were performed using Prism version 10.1.2 or newer (GraphPad).

## Supplementary Material

Supplementary materials

Supplementary tables

Data file S1

MDAR Reproducibility Checklist

## Data Availability

All data associated with this study are present in the paper or supplementary materials. The mass spectrometry data disclosed in this manuscript have been deposited on the ProteomeXchange and can be accessed with identifier “PASS03798.” Plasmids and cell lines are available by request under a material transfer agreement with Johns Hopkins University by contacting sbzhou@jhmi.edu. Transcriptome data are deposited in the Gene Expression Omnibus with accession number GSE266456.
