# Supplementary material for: Co-STARs combine the advantages of chimeric antigen and T-cell receptors for the treatment of tumors with low antigen densities
Source: Sci Transl Med. Author manuscript; Available in PMC 2025 Jul 4. (PMC12226805; doi:10.1126/scitranslmed.adg7123)
Supplement: Supplementary materials [file NIHMS2090446-supplement-Supplementary_materials.pdf]

Science Translational Medicine
AAAS

Supplementary Materials for

Preclinical studies show that Co-STARs combine the advantages of chimeric antigen and T cell receptors for the treatment of tumors with low antigen densities

Brian J. Mog *et al.*

Corresponding author: Bert Vogelstein, bertvog@gmail.com; Shibin Zhou, sbzhou@jhmi.edu

Sci. Transl. Med. **16**, eadg7123 (2024)
DOI: 10.1126/scitranslmed.adg7123

The PDF file includes:

Materials and Methods
Figs. S1 to S19
References (*105–112*)

Other Supplementary Material for this manuscript includes the following:

Tables S1 to S6
Data file S1
MDAR Reproducibility Checklist

Materials and Methods

Cell lines

KMS26 was obtained from the Japanese Collection of Research Bioresources Cell Bank (JCRB). NALM6 cells virally transduced with GFP and luciferase were generously provided by Marty Pomper (*105*). T2 cells were obtained from American Type Culture Collection (ATCC). KMS26, NALM6, and T2 cells were cultured in RPMI-1640 (ATCC, 30-2001) with 10% FBS (Cytiva, SH30070.03) and 1% penicillin-streptomycin (Thermo Fisher Scientific, 15140163). Cells were grown in a humidified incubator at 37°C in 5% CO_2. The KMS26-WT (parental) and KMS26-NULL (*TP53* KO) cell lines were modified with GFP and luciferase as previously described (*39*).

Generation of NALM6-MUT cell line

The IDT Alt-R CRISPR system (IDT) was used to convert the wildtype *TP53* allele of NALM6 to the R175H mutant to generate the NALM6-MUT cell line. A homology directed repair template (HDRT) encoded the R175H mutation as well as 5 synonymous mutations to abrogate the PAM site and prevent template switching during repair (*106*). A *TP53* targeted Cas9 crRNA was duplexed with tracrRNA at a 1:1 molar ratio at 95 °C for 5 mins. After cooling to room temperature, 100 pmols of cr:tracrRNA duplex was complexed with 50 pmol of Cas9 nuclease for 15 minutes at room temperature. The resulting ribonucleoprotein was mixed with 60 pmol of a single stranded HDRT (IDT, Alt-R HDR Donor Oligo) encoding the R175H mutation and 5 x 10^5 NALM6 cells in 20 μl of OptiMEM (Thermo Fisher Scientific, 31985062) in a 0.1 cm electroporation cuvette (Bio-Rad, 1652089). The mixture was then electroporated at 100 V for 10 ms using an ECM 2001 (BTX). Cells were immediately recovered with warm culture medium. After 1 week of culture, the electroporated cells were plated by limiting dilution. Individual clones were screened by sanger sequencing a PCR amplicon encompassing the edit

site to assess the *TP53* R175H mutation status. Sequences of the gRNA, HDRT, and primers are included in supplementary table S4.

Detection and Quantification of p53RH antigen

Neoantigen detection and quantification was performed through Valid-NEO pipeline by Complete Omics as previously described (*38, 39, 107*). In brief, a total of 500 million cells were lysed and pHLA complexes were immunoprecipitated using Valid-NEO enrichment column (Complete Omics Inc) packed by matrix conjugated with anti-human HLA-A, B, C antibody clone W6/32 (BioXcell, BE0079). After elution, dissociation, filtration, size exclusion separation, cleanup and fractionation procedures, peptides were subsequently analyzed on a triple quadrupole mass spectrometer. Transition parameters were manually examined and curated to exclude ions with excessive noise due to co-elution with impurities. Absolute copy numbers of neoantigen peptides presented on the cell surface were calculated based on the Valid-NEO quantification using the AQUA heavy isotope labeled peptides as previously described (*107*).

Flow cytometry

Flow cytometry was performed with an IntelliCyt iQue Screener PLUS (Sartorius). Cells were labeled with a viability dye, either LIVE/DEAD Fixable Near-IR Dead Cell Stain (Thermo Fisher Scientific, L34975) or LIVE/DEAD Fixable Violet Dead Cell Stain (Thermo Fisher Scientific, L34955). To assess HLA-A*02:01 expression, NALM6 isogenics were stained with Brilliant Violet (BV)-785 anti-human HLA-A*02 (BB7.2, BioLegend, 343328) or a mouse isotype IgG2b, κ (HI100, BioLegend, 304140). T cells were stained with combinations of the following anti-human antibodies: APC-CD3 (SK7, BioLegend, 344812), BV421-CD3 (SK7, BioLegend, 344834), BV785-CD4 (RPA-T4, BioLegend, 300554), PE-Vio770-CD4 (M-T466,

Miltenyi, 130-113-255), APC-CD2 (RPA-2.10, BioLegend, 300214), BV421-CD2 (RPA-2.10, BioLegend, 300230), APC-NGFR (ME20.4, BioLegend, 345108) , BV785-PD-1 (EH12.1, BD, 563789), BV605-LAG-3 (11C3C65, BioLegend, 369316), or PE-dazzle/594-TIM-3 (F38-2E2, BioLegend, 345034). CD8+ T cell populations were defined as T cells that were not stained with CD4-targeted antibodies when included. The expression of TCR, CAR, or CAR-derivatives was assessed with PE, APC, or BV421 labeled p53RH tetramer (FHCRC Immune Monitoring Core facility), anti-mouse TRBC (H57-597, BioLegend, 109230), or anti-human Vβ21.3 (REA894, Miltenyi, 130-114-839). When staining T cells in peripheral blood of mice, a BV605 labeled anti-murine CD45 antibody (30-F11, BioLegend, 103140) and TruStain FcX (anti-mouse CD16/32) antibody (93, BioLegend, 101320) were also used. Flow cytometry data was analyzed with either the iQue Forecyt Software (Sartorius) or FlowJo v.10.1 software (BD).

Peptide pulsing

T2 cells were washed with serum-free RPMI-1640 media, then incubated at 1-2 x 10^6 cells/mL with 10 µg/mL beta-2 microglobulin (ProSpec, PRO-337) and specified concentrations of the p53RH peptide (HMTEVVRHC, Peptide 2.0). Pulsed cells were incubated on low attachment plates (Corning, 3471) in a 37 °C incubator for at least 4 hrs. Prior to use in functional assays, pulsed cells were washed 2 times with serum containing media.

In vitro short-term co-culture assays

T cell knock-in (KI) frequency was normalized to the lowest frequency by TCR-Control T cells. Then a specified number of KI+ T cells were mixed with a specified number of cancer cells (generally 1 x10^4 KI+ T cells with 5 x10^4 cancer cells unless otherwise specified in the figure legend) in 200 µl of RPMI-1640 media supplemented with 10% FBS, 1% PS, and 100 IU/mL

IL-2. Co-cultures were incubated from 18-24 hrs in a 37 °C incubator, as specified in figure legends. At the termination of the assay, 100 µl conditioned supernatant was used for the Human IFN-γ Quantikine Kit (R&D Systems, PDIF50C), the MILLIPLEX Luminex assay (Millipore-Sigma, HCD8MAG-15K), or a custom Luminex discovery assay (R&D Systems, LXSAHM) per the manufacturers' instructions. A bioluminescence cytotoxicity assay (Promega, E2510) was performed per the manufacturer's instructions on the remaining 100 µl containing the cells. Cytotoxicity was calculated by normalizing to the TCR-Control luminescence signal: [1 – (experimental well)/(TCR-Control well)] x 100.

<u>Multiple stimulation assays</u>

When using live-cell imaging to assess cytotoxicity, 1 x 10^4 KI+ T cells (knock-in frequency normalized to the lowest frequency by TCR-Control T cells) were mixed with 5 x10^4 NALM6-MUT cells in 200 µl of cytokine-free RPMI-1640 media supplemented with 10% FBS and 1% PS. Live cell imaging with an Incucyte SX5 (Sartorius) was used to quantify the number of GFP+ NALM6-MUT cells every 3-4 hrs. After approximately 48 hrs, 100 µl of conditioned supernatant was removed and 5 x 10^4 NALM6-MUT cells in 100 µl of fresh media were added to the co-culture. This process was repeated for 24 days. At the end of the assay, the number of living KI+ T cells and NALM6-MUT cells were quantified by flow cytometry.

When using flow-cytometry assessment of cytotoxicity at each time point, 4 x 10^3 KI+ T cells (knock-in frequency normalized to the lowest frequency by TCR-Control T cells) were labeled with cell-trace-violet and were mixed with 1.6 x10^4 KMS26-MUT cells in v-bottom plates in 3 replicates per timepoint. After 48 hours, the number of T cells and KMS26-MUT cells were quantified by flow cytometry. Then 3.2 x10^4 KMS26-MUT cells were added to the remaining replicates. This process was repeated for 2 additional stimulations.

Bulk RNAseq and Analysis

STAR-3 and Co-STAR-1 T cells were generated using CRISPR-HDR as described above. On days 8-9 after activation, knock-in+ T cells were isolated using the EasySep Release Human PSC-Derived Neural Crest Cell Positive Selection Kit (StemCell, 100-0047). On days 13-14, T cells were cocultured with KMS26-MUT, KMS26-NULL, or no target cells at an E:T ratio of 1:5 for 18 hours followed by flow sorting of CD4+ and CD8+ T cells into RNAprotect Tissue Reagent (Qiagen, 76104). Total RNA was isolated with the RNeasy Mini Kit (Qiagen, 74104) according to the manufacturer's instructions and quantified by RNA ScreenTape (Agilent, 5067-5576). The SMART-Seq mRNA LP (with UMIs) kit (Takara, 634765) and the Unique Dual Index Kit (1–96) (Takara, 634752) were used to generate cDNA and prepare sequencing libraries from two independent batches of T cells, with 3 technical replicates per condition. Libraries were sequenced on a NovaSeq 6000 (Illumina) using the 100 cycle single-end configuration at approximately 25 million reads per technical replicate.

FASTQ files were demultiplexed and only reads containing the 5' unique molecular identifiers (UMIs) were retained after trimming adapters and extracting the UMI. Reads were aligned to hg38 with RNA STAR (*108*) followed by UMI deduplication using UMI-tools (*109*). Gene-counts of unique reads were generated with HTseq (*110*). Differentially expressed genes were identified with DEseq2 and defined as genes with adjusted p-value less than 0.05, base-mean expression greater than 4, and absolute value of fold change greater than 1.2 (*111*). Heatmaps were Z-score scaled by row on variance stabilizing transformations of the normalized count matrix produced by DEseq2. Pathway analysis was performed using the STRING tool on differentially expressed genes and Reactome gene sets were reported (*112*). The strength of enrichment was calculated with the following expression: $\log_{10}$(observed number of genes/expected number of genes).

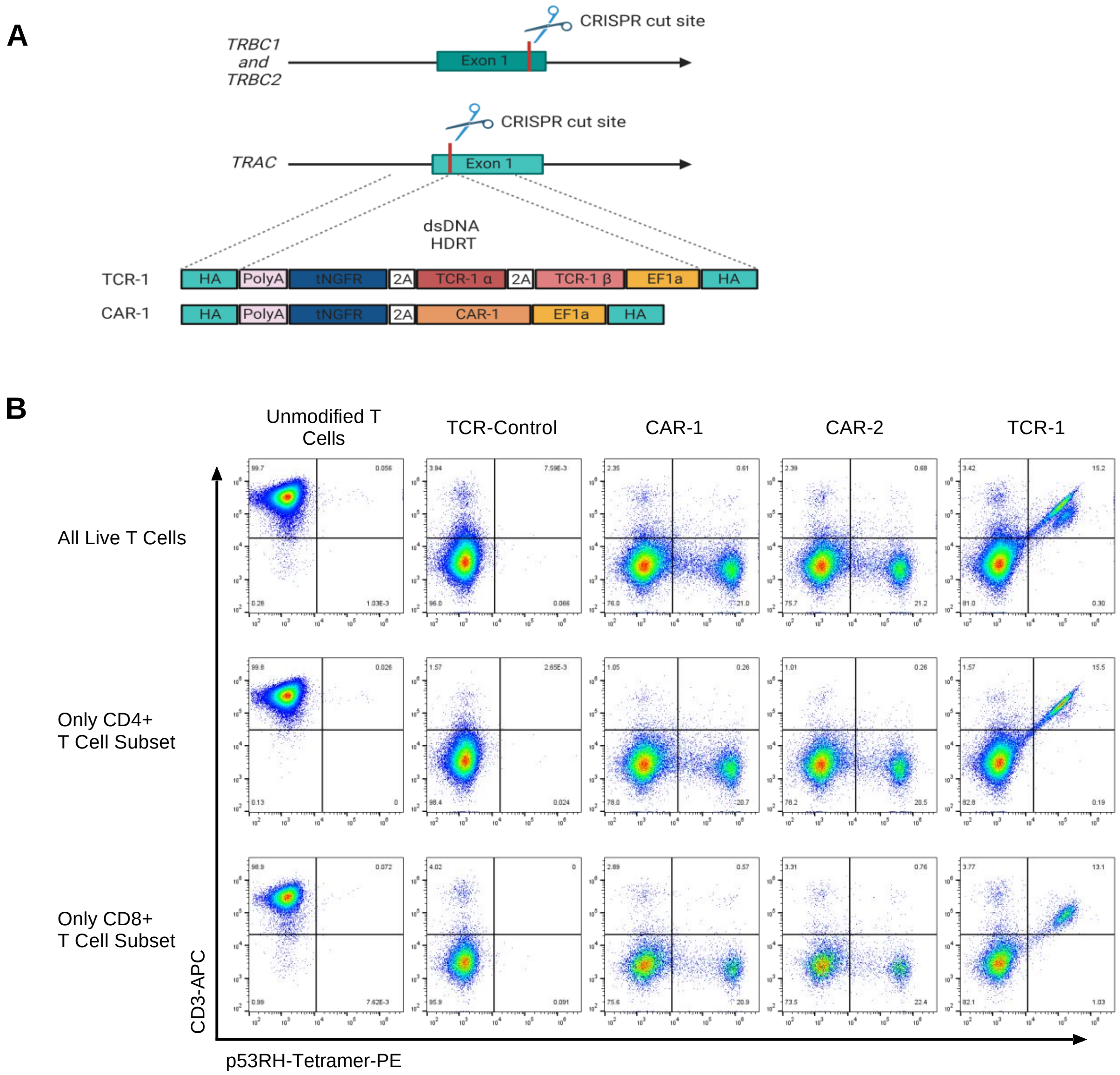

Fig. S1. CRISPR knock-in and knock-out strategy in primary human T cells.

(A) Diagram showing the simultaneous *TRAC* knock-in (KI) and *TRBC1* and *TRBC2* knock-out (KO) approach. TCR-1 and CAR-1 homology directed repair templates (HDRTs) are shown specifically. Double stranded DNA (dsDNA) HDRTs include an EF1α promoter (EF1α), the receptor domain(s), a tNGFR tag, and a simian virus 40 polyadenylation signal following the stop codon of the tNGFR tag. Independent proteins are separated by furin-2A sequences (2A). Homology arms (HAs) are approximately 300 base pairs (bps) in length. (B) Flow cytometric

characterization of modified T cells with the p53RH tetramer and CD3 staining reagents four days after nucleofection. The top row displays all live T cells, while the middle row displays the CD4+ T cell subset and the bottom row displays the CD8+ T cell subset (defined as CD4 staining negative).

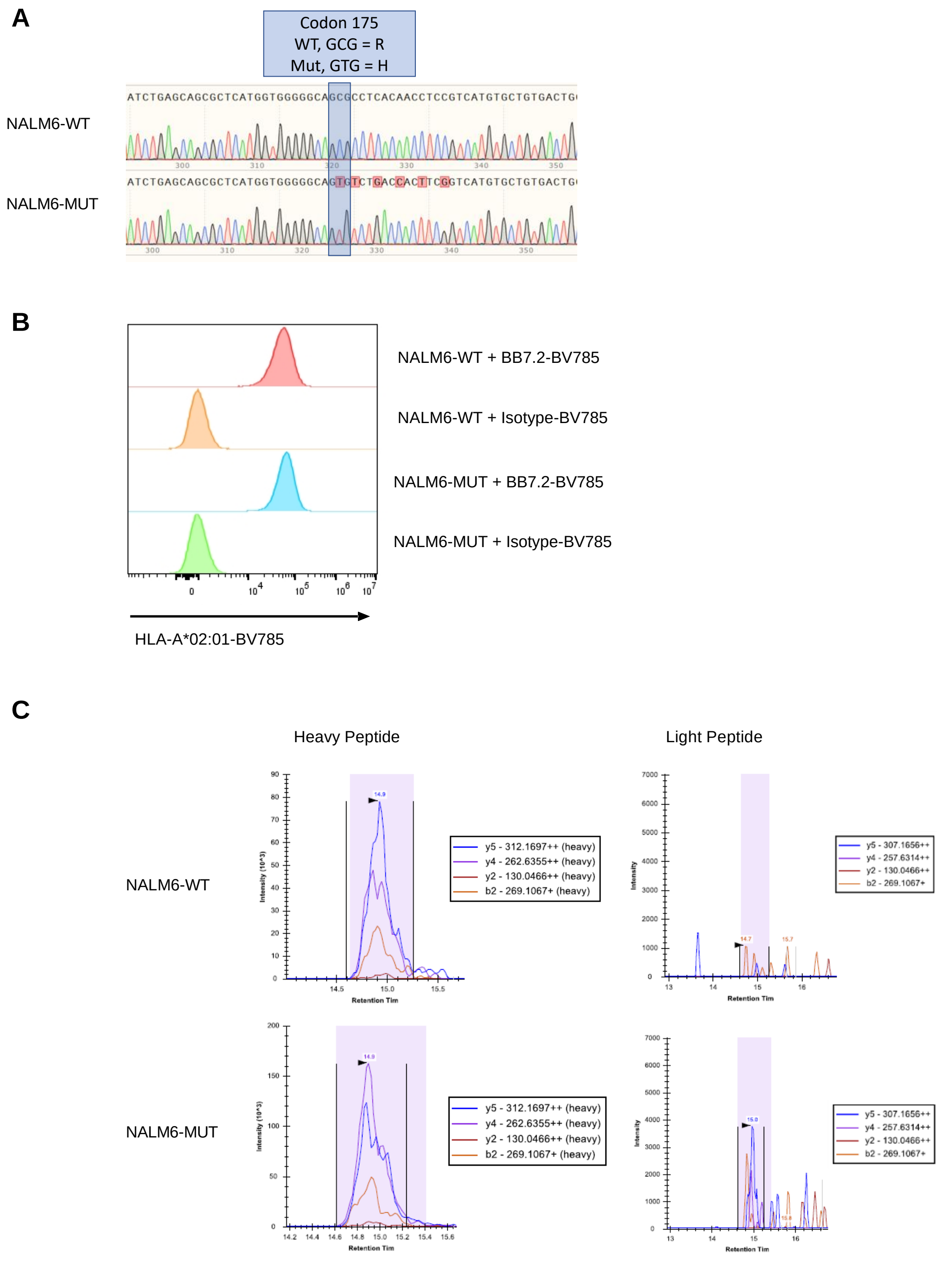

Fig. S2. Generation of the NALM6-MUT cell line.

(A) Sanger sequencing of the *TP53* locus near codon 175 of both the NALM6-WT line and the NALM6-MUT line. The HDRT included five synonymous mutations between the substitution encoding the R175H mutation and the CRISPR Cas9 cut site to minimize template switching

during double strand break repair. “GCG” listed in the figure is the antisense codon of “CGC” encoding arginine, and “GTG” is the antisense codon of “CAC” encoding histidine. (B) Flow cytometric staining for HLA-A*02:01 with BB7.2, an antibody for HLA-A*02:01, or an isotype control antibody labeled with brilliant violet 785 on the NALM6-WT and NALM6-MUT lines. (C) Mass spectrometry quantification of the p53RH peptide (HMTEVVRHC) eluted from the HLA molecules of the NALM6-WT and NALM6-MUT lines. Colored lines indicate ions fragmented from the targeted peptide with m/Z values listed in the tables next to each graph.

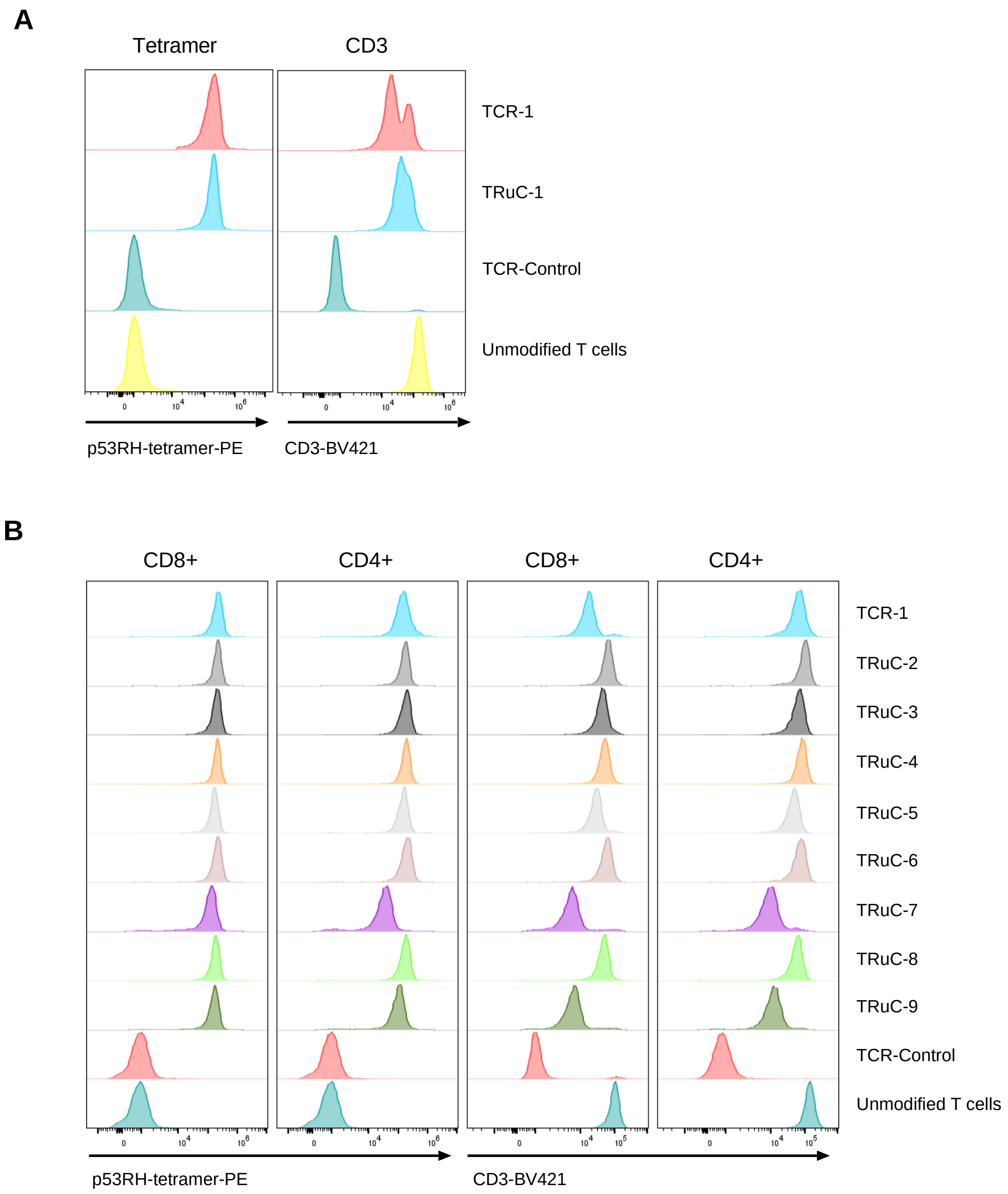

Fig. S3. Flow cytometric characterization of Tier 2 TRuC T cells.

(A) Flow cytometric staining of tNGFR+ modified T cells with p53RH tetramer labeled with phycoerythrin (p53RH-tetramer-PE) and SK7, an antibody for CD3, labeled with brilliant violet 421 (CD3-BV421) ten days after nucleofection. (B) Comparison of p53RH tetramer and anti-CD3 staining on tNGFR+ CD4+ and tNGFR+ CD8+ T cells 11 days after nucleofection. TCR-Control and Unmodified T cells show all live, single cells (A and B).

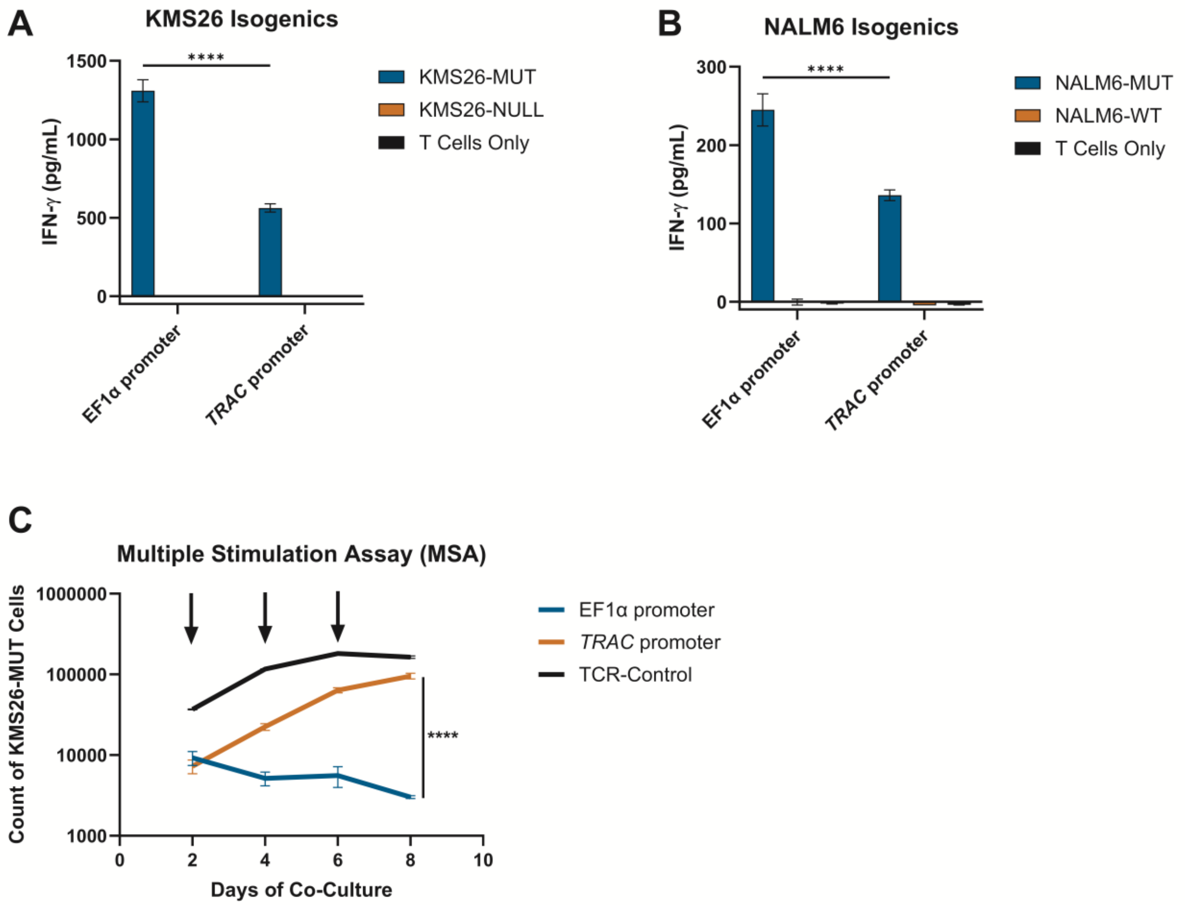

Fig. S4. Comparison of endogenous *TRAC* promoter with exogenous EF1α promoter for expression of STAR-3.

CRISPR strategies were designed to insert a STAR-3 construct at the *TRAC* locus either under control of the endogenous *TRAC* promoter or with an exogenous EF1α promoter. The endogenous *TRAC* promoter strategy utilized a Cas9 ribonucleoprotein (RNP) while the exogenous EF1α promoter strategy utilized a Cpf1 RNP. Both Cas9 and Cpf1 RNPs targeted overlapping regions of exon 1 of the *TRAC* locus and included concomitant KO of the *TRBC* loci. For this experiment, the STAR-3 construct utilized human TCR constant domains as opposed to the murine constant domains used in all other experiments. (A) Modified T cells were cultured with the KMS26 cell set at an E:T ratio of 1:5 for 20 hrs. Conditioned supernatant was assayed for IFN-γ by ELISA. (B) Co-culture conditions match those of A but the NALM6

isogenic set was used. For A and B, data are shown as means ± SD of three technical replicates, except for the T Cells Only conditions, which are two technical replicates. (C) Modified T cells (4×10^3) labeled with Cell Trace Violet were incubated with GFP-expressing KMS26-MUT cells (1.6×10^4) in 16 replicates. Every 48 hrs, the number of cancer cells in four replicates was quantified by flow cytometry and additional KMS26-MUT cells (3.2×10^4) were added to the remaining replicates. This process was repeated 3 more times for a total of 4 timepoints. Data are shown as means ± SD of four technical replicates. ****$P < 0.0001$ by two-way ANOVA with Tukey's multiple comparison test for all panels. Data in this figure are from N = 1 experiment.

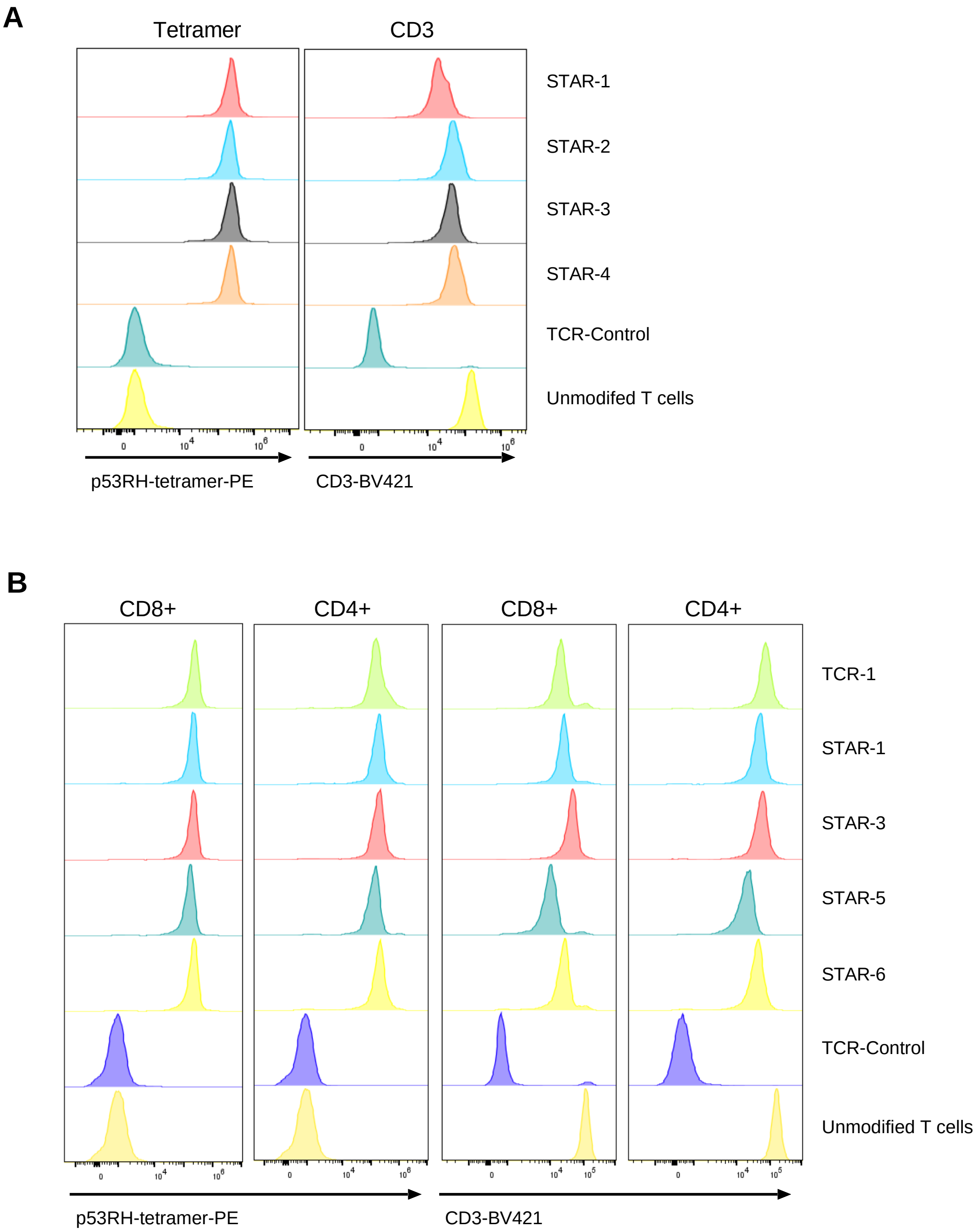

Fig. S5. Flow cytometric characterization of Tier 3 STAR T cells.

(A) Flow cytometric staining of tNGFR+ modified T cells with p53RH tetramer and anti-CD3 ten days after nucleofection. (B) Comparison of p53RH tetramer and anti-CD3 staining on tNGFR+ CD4+ and tNGFR+ CD8+ T cells 11 days after nucleofection. TCR-Control and Unmodified T cells show all live, single cells (A and B).

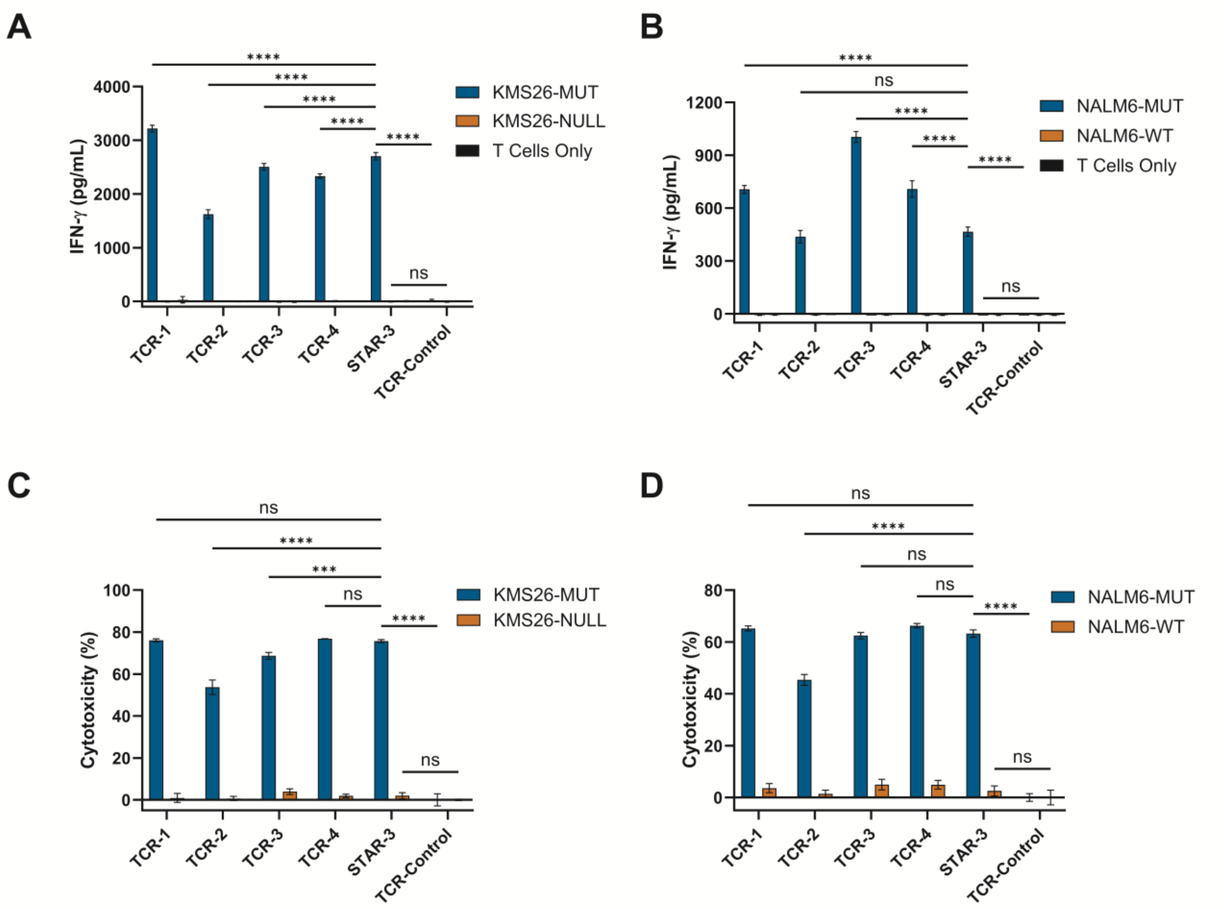

Fig. S6. Comparing STAR-3 to four p53RH reactive TCRs.

(A) Modified T cells were cultured with the KMS26 isogenic cell set at an E:T ratio of 1:5 for 20 hrs. Conditioned supernatant was assayed for IFN-γ by ELISA. (B) Co-culture conditions matched those of A except the NALM6 isogenic cell set was used. (C) The cytotoxicity of modified T cells in the same co-culture described in A was quantified by bioluminescence. (D) The cytotoxicity of modified T cells in the same co-culture described in B was quantified by bioluminescence. Data are shown as means ± SD of three technical replicates, except for the T Cells Only conditions, which are two technical replicates. Data are representative of N = 2 independent experiments and n = 2 healthy donors. ****$P < 0.0001$, ***$P < 0.001$, ns (not significant) by two-way ANOVA with Tukey’s multiple comparison test for all panels.

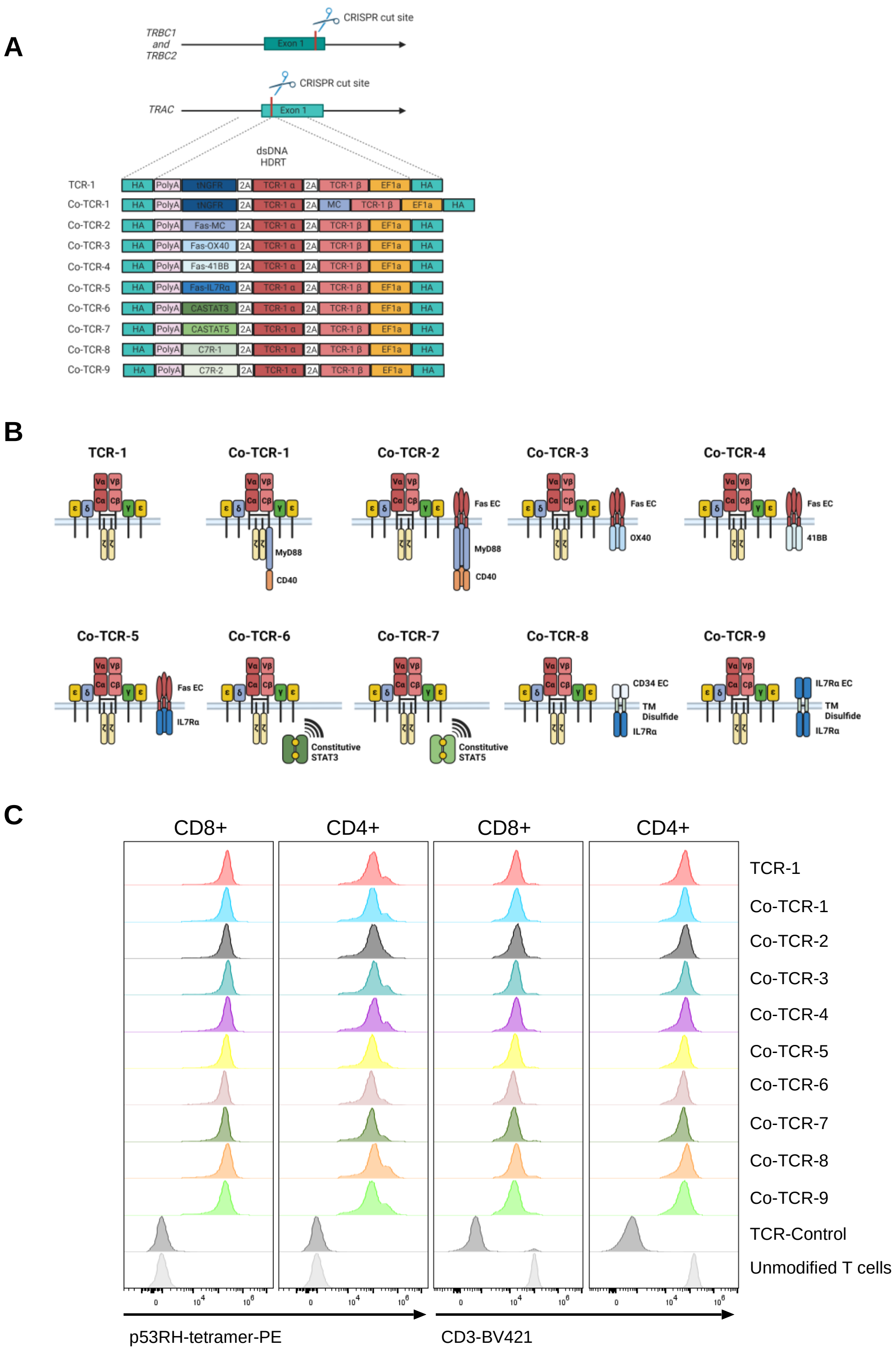

Fig S7. Screening approach for candidate co-stimulatory domains.

(A) Schematic showing the *TRAC* knock-in and *TRBC* knock-out strategy for testing multiple co-stimulatory constructs alongside TCR-1. When the co-stimulatory construct encodes an

independent protein domain, the tNGFR domain is replaced by the co-stimulatory sequence. For this screen, the TCR-1 construct encodes the human TCRα and TCRβ constant domains. (B) Diagrams depicting each of the nine co-stimulatory designs tested. For Co-TCR-1, the MC domains are connected to the intracellular side of the TCRβ transmembrane domain. Co-TCR-2 through -5 link the Fas extracellular (Fas EC) and transmembrane domains to MC, OX40, 4-1BB, and IL7Rα signaling domains. Co-TCR-6 and -7 co-express STAT3 (CASTAT3) and STAT5 (CASTAT5) molecules bearing mutations which impart constitutive activity. Co-TCR-8 and -9 co-express the IL7Rα intracellular domain linked to the IL7Rα transmembrane domain with a CPT insertion which drives dimerization and constitutive activity. Co-TCR-8 utilizes a CD34 extracellular domain (CD34 EC) and Co-TCR-9 utilizes the IL7Rα extracellular domain (IL7Rα EC). (C) Flow cytometric quantification of p53RH and anti-CD3 staining in CD8+ tetramer+ and CD4+ tetramer+ T cells ten days after nucleofection. TCR-Control and Unmodified T cells show all live, single cells.

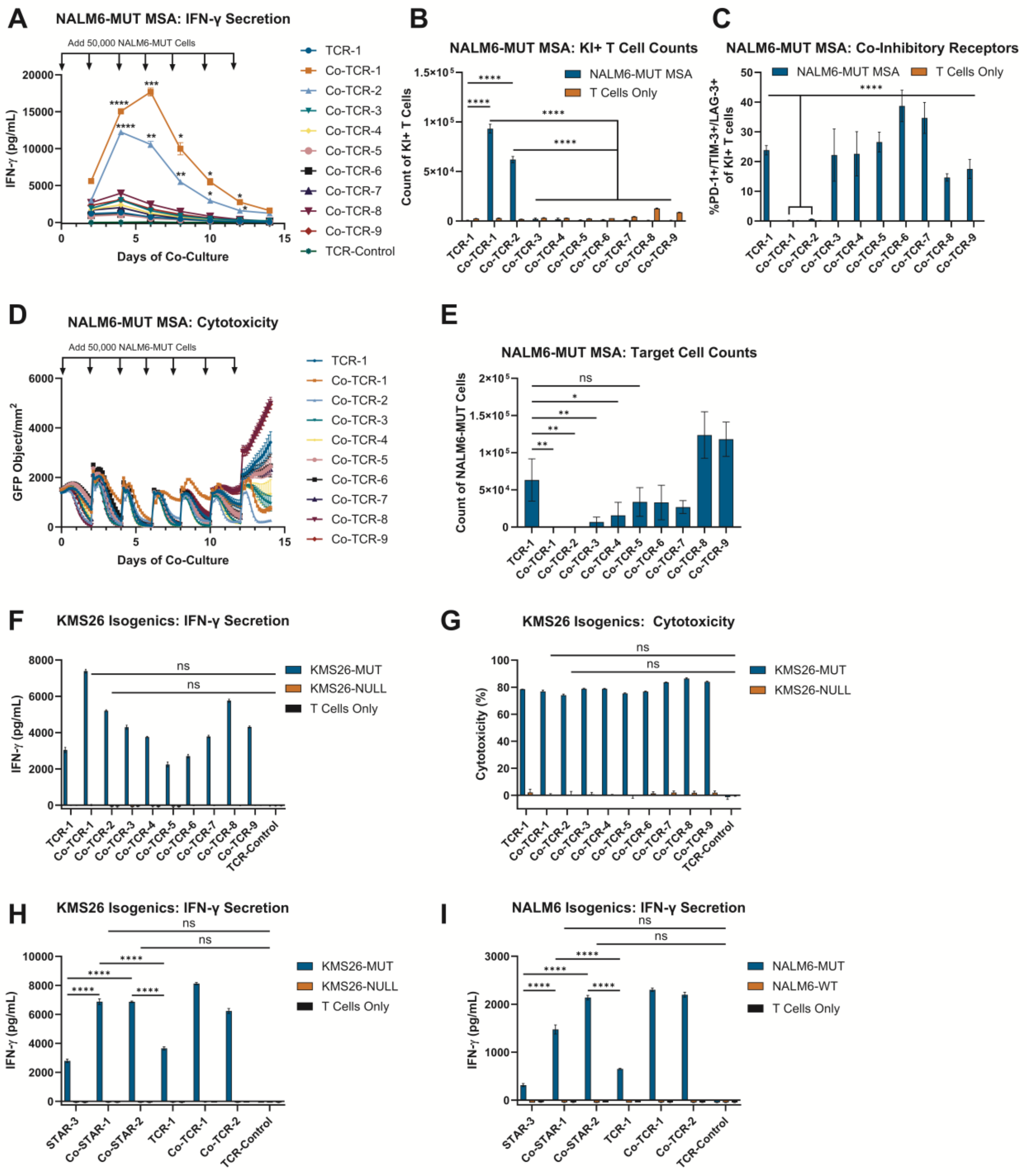

Fig. S8. Functional assessment of co-stimulatory candidates.

(A) NALM6-MUT cells (5 x 10^4) were co-incubated with modified T cells (1 x 10^4) with exogenous IL-2. Every 48 hrs, 5 x 10^4 NALM6-MUT cells were added to the co-culture with additional IL-2 (multi-stimulation assay or MSA). Conditioned supernatant collected every two

days was assayed for IFN-γ by ELISA. Data shown are means ± SD of three technical replicates. (B) Flow cytometric quantification of knock-in positive (KI+) T cell numbers at the end of the MSA in A. (C) Flow cytometric quantification of co-inhibitory receptor expression on KI+ T cells at the end of the MSA show in A. (D) Live cell imaging was used to quantify cancer cell growth during the assay described in A. Data are representative of means ± SEM of four technical replicates. (E) Flow cytometric quantification of NALM6-MUT cells at the end of the MSA in A. Data in B, C, and E show means ± SD of four technical replicates. (F) Modified T cells (1 x 10^4) were incubated with the KMS26 isogenic cell set at an E:T ratio of 1:5 for 20 hrs. IFN-γ in conditioned supernatant was measured by ELISA. (G) Cytotoxicity from the co-culture described in F was measured by a bioluminescence assay. Data in F and G show means ± SD of three technical replicates, except for T Cells Only, which have only two technical replicates. (H) Modified T cells (1 x 10^4) were incubated with the KMS26 isogenic cell set (5 x 10^4) for 24 hrs. Conditioned supernatant was analyzed for IFN-γ by ELISA. (I) Co-culture conditions match those in H except the NALM6 isogenic cell set was used. Data in H and I are shown as means ± SD of three technical replicates and represent N = 3 independent experiments and n = 2 healthy donors. Constructs in A-G were designed with human TCRα and TCRβ constant domains, while constructs in H and I used modified murine constant domains as described in the Methods section. ****$P < 0.0001$, ***$P < 0.001$, **$P < 0.01$, *$P < 0.05$, ns (not significant) by two-way ANOVA with Tukey's multiple comparison test for all panels except E for which a one-way ANOVA with Tukey's multiple comparison was used.

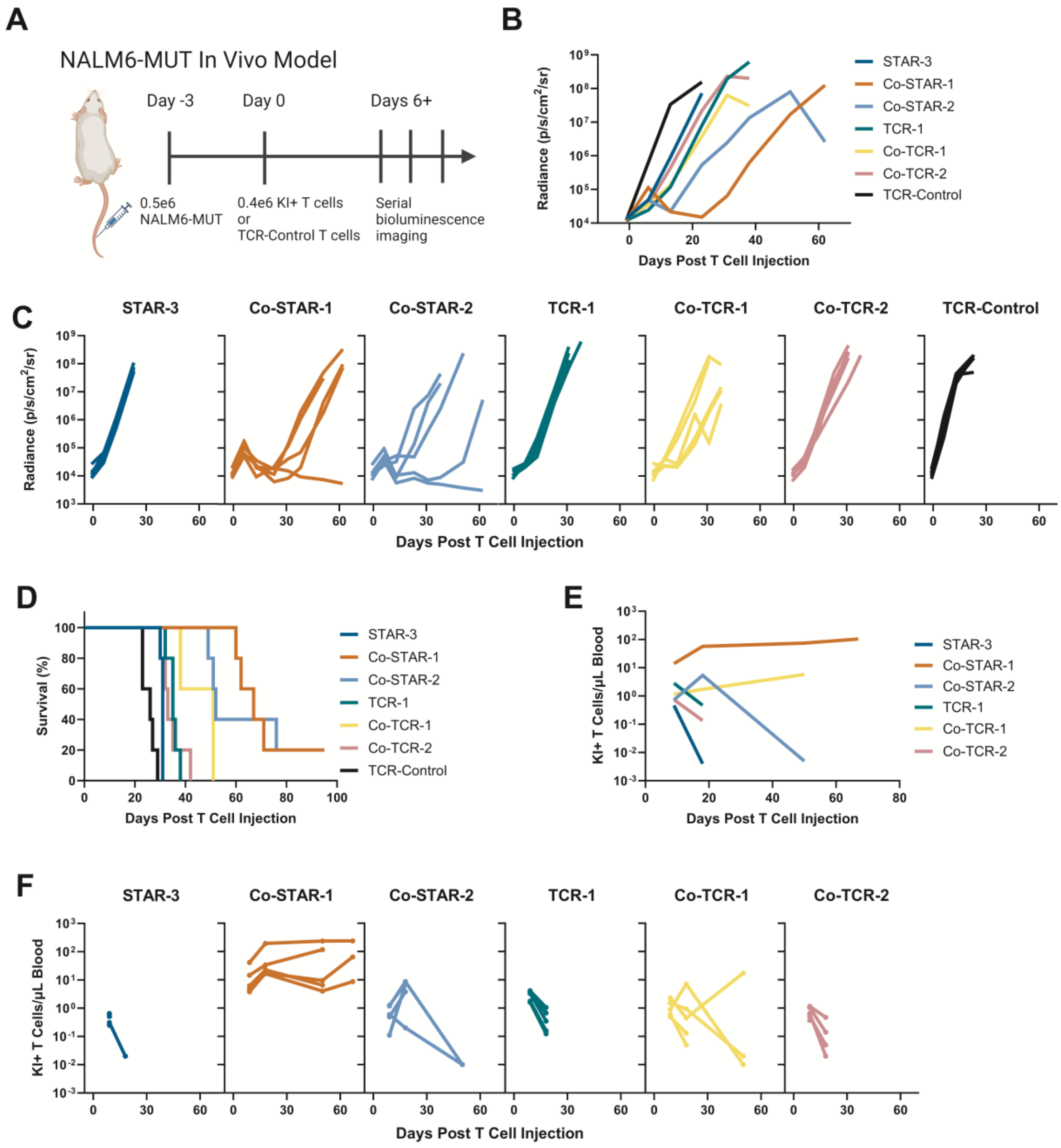

Fig. S9. NALM6-MUT in vivo model.

(A) Schematic showing the NALM6-MUT in vivo model. NSG mice were inoculated through the tail vein with 0.5 x 10^6 NALM6-MUT cells on day -3 followed by randomization based on BLI signal on day -1. Tail vein injection of modified T cells (either 0.4 x 10^6 KI+ T cells normalized to 13.6% KI frequency with TCR-Control T cells or 2.9 x 10^6 TCR-Control T Cells) was performed on day 0. Approximately weekly BLI imaging was used to track cancer cell

growth. (B) Radiance measurements for treatment groups are displayed as means. Curves are truncated when all mice in a treatment arm died before the specified timepoint. BLI measurements were discontinued 62 days after initial treatment. Two-way ANOVA with Holm-Šídák multiple comparison correction was used to compare all treatment groups of the initial 7 with at least 4 surviving mice through day 31 ($P < 0.0001$ comparing Co-STAR-2 to Co-TCR-2) and through day 38 ($P < 0.001$ comparing Co-STAR-1 to Co-TCR-1; $P > 0.05$ comparing Co-STAR-1 to Co-STAR-2). (C) The same radiance data in B are displayed for individual mice by treatment group. (D) Kaplan-Meier survival curves of seven treatment groups, n=5 mice per group. Log-rank Mantel-Cox test with Bonferroni-Holm correction was used ($P < 0.05$ comparing Co-STAR-1 to TCR-1, Co-TCR-1, and Co-TCR-2; $P > 0.05$ comparing Co-STAR-1 and Co-STAR-2). (E) Quantification of KI+ T cells in peripheral blood of mice using flow cytometry. Measurements are shown as means, n = 5 mice per treatment group. The curves are truncated either when all mice within a treatment group died or when the number of KI+ T cells detected in peripheral blood by flow cytometry was zero. Two-way ANOVA with Holm-Šídák multiple comparison correction was used to compare all treatment groups through day 18 ($P < 0.01$ comparing Co-STAR-1 to all other groups). (F) The same KI+ T cell concentrations from E are displayed for individual mice by treatment group. Data in this figure were from N = 1 experiment.

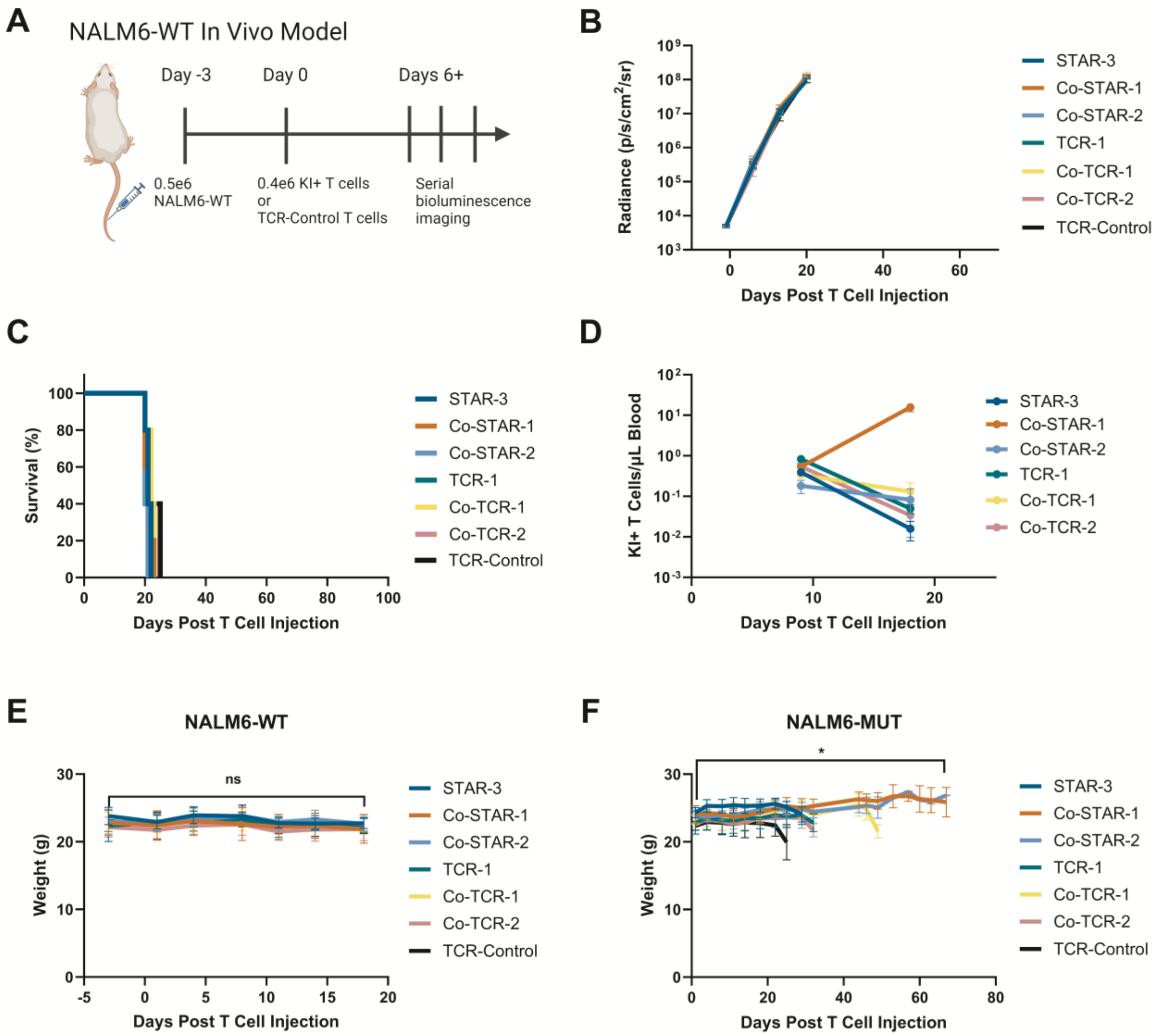

Fig. S10. NALM6-WT in vivo model.

(A) Schematic showing the NALM6-WT in vivo model. NSG mice were inoculated through the tail vein with 0.5 x 10^6 NALM6-WT cells on day -3 followed by randomization on day -1 based on BLI signal. Tail vein injection of modified T cells (either 0.4 x 10^6 KI+ T cells normalized to 8.3% KI frequency with TCR-Control T cells or 4.8 x 10^6 TCR-Control T Cells) was performed on day 0. (B) Approximately weekly BLI imaging was used to track cancer cell growth. Data are shown as means. Two-way ANOVA with Holm-Šídák multiple comparison correction was used to compare all treatment groups ($P > 0.05$ for all comparisons). (C) Kaplan-Meier survival

curves of seven treatment groups, n=5 mice per group. Log-rank Mantel-Cox test with Bonferroni-Holm correction was used (*P* > 0.05 for all comparisons). (D) Flow cytometric quantification of KI+ T cells detected in peripheral blood of mice from each treatment group. Data are shown as means ± SEM, n = 5 mice per treatment group. Two-way ANOVA with Holm-Šídák multiple comparison correction was used (*P* < 0.0001 comparing Co-STAR-1 to all other groups). (E) Weight measurements of all mice in the NALM6-WT experiment. (F) Weight measurements of all mice in the NALM6-MUT experiment. For E and F, data are plotted as means ± SD of values from five mice and curves are truncated when 4 or more mice in a treatment group have died. ****$P < 0.001$, *$P < 0.05$, ns (not significant) by two-way ANOVA with Dunnett's multiple comparison test comparing the initial timepoint to each subsequent time point for all treatment groups (E and F). Data in this figure were from N = 1 experiment.

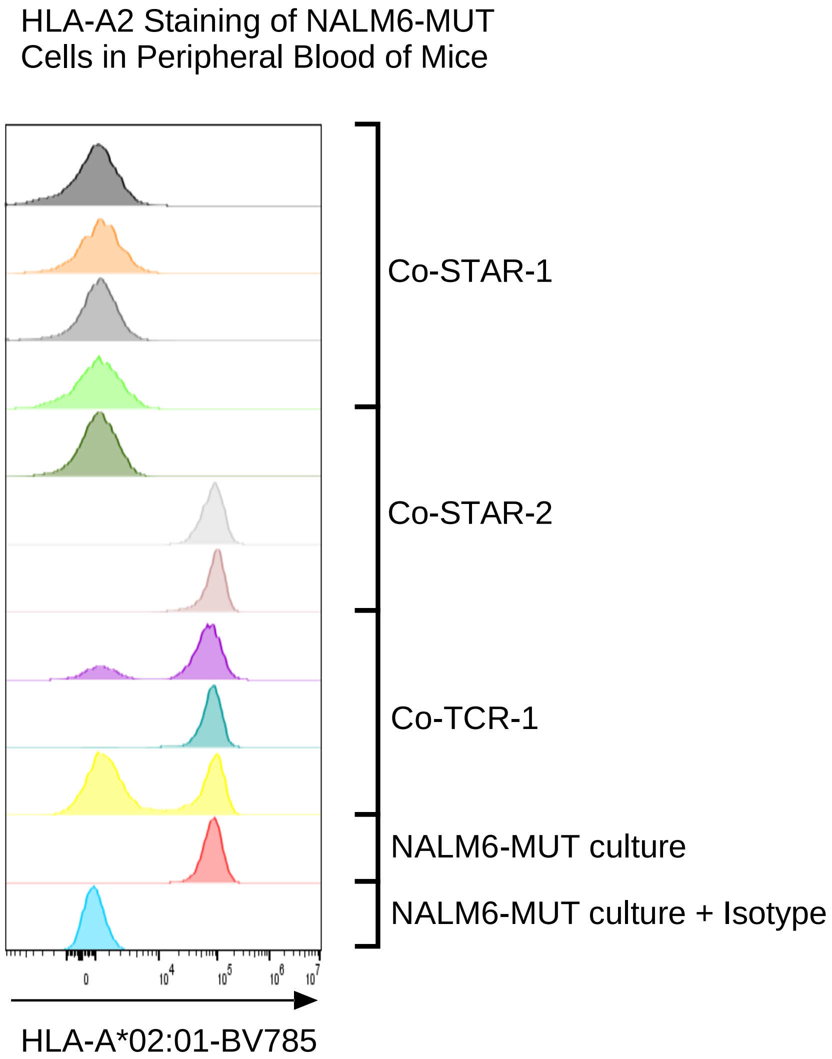

Fig. S11. HLA-A*02:01 expression on cancer cells in NALM6-MUT in vivo model.

Flow cytometric staining of HLA-A*02:01 on NALM6-MUT cells detected in peripheral blood of mice on days 50 or 67 after T cell injection. Each row shows live NALM6-MUT cells from a different mouse or from in vitro culture.

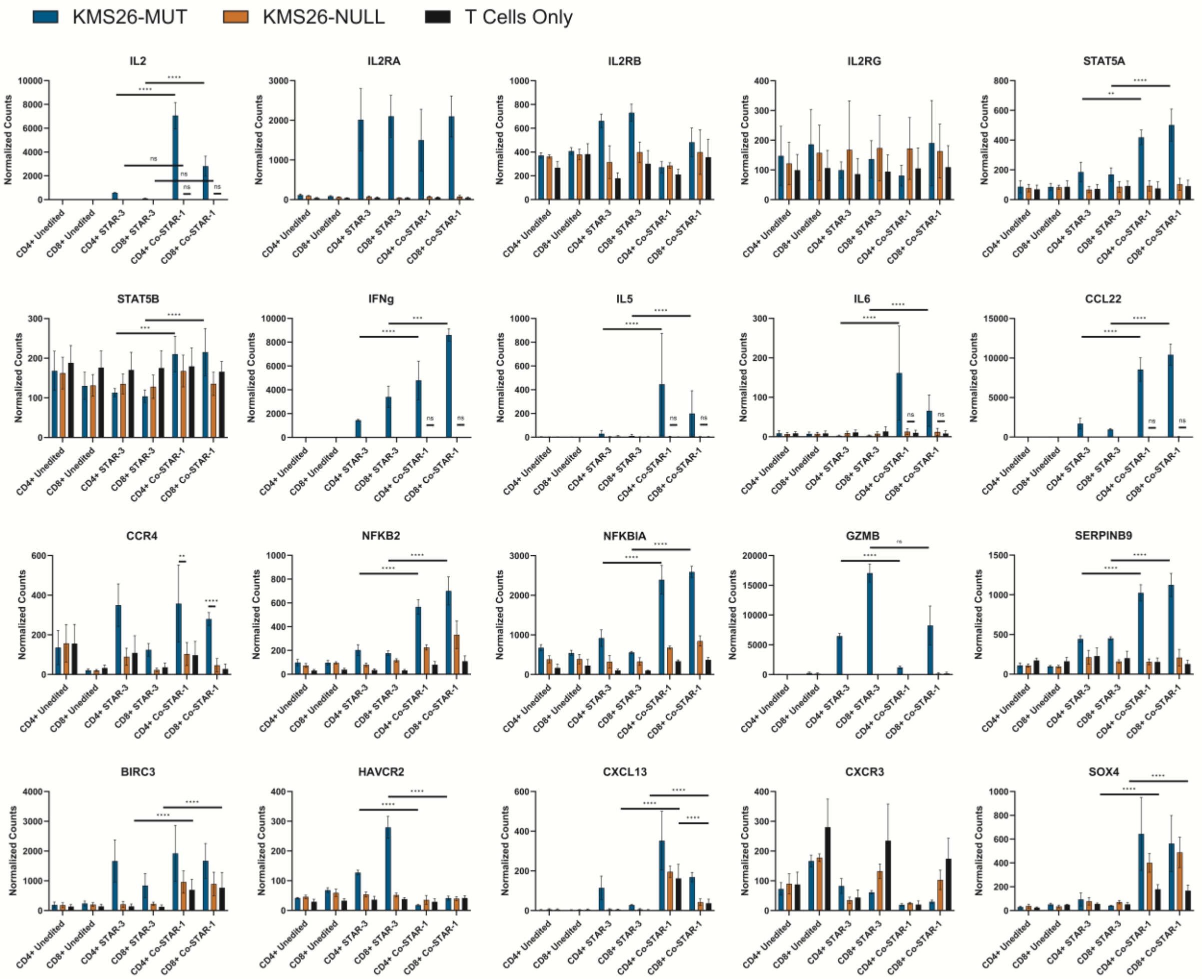

Fig. S12. Normalized counts of selected genes modulated in Co-STAR-1 T cells.

Co-STAR-1, STAR-3 and unmodified T cells were incubated with KMS26-MUT, KMS26-NULL, or no target cells at an E:T ratio of 1:5 for 18 hrs in the absence of exogenous IL-2 followed by flow sorting and transcriptomic analysis of CD4+ and CD8+ T cells. Normalized transcript counts from the DEseq2 analysis for specific genes are displayed. Data show means ± SD of 6 replicates. ****p-*adjusted* < 0.0001, ***p-adjusted* < 0.01, **p-adjusted* < 0.05, ns (not significant) by the Wald test of the DEseq2 analysis.

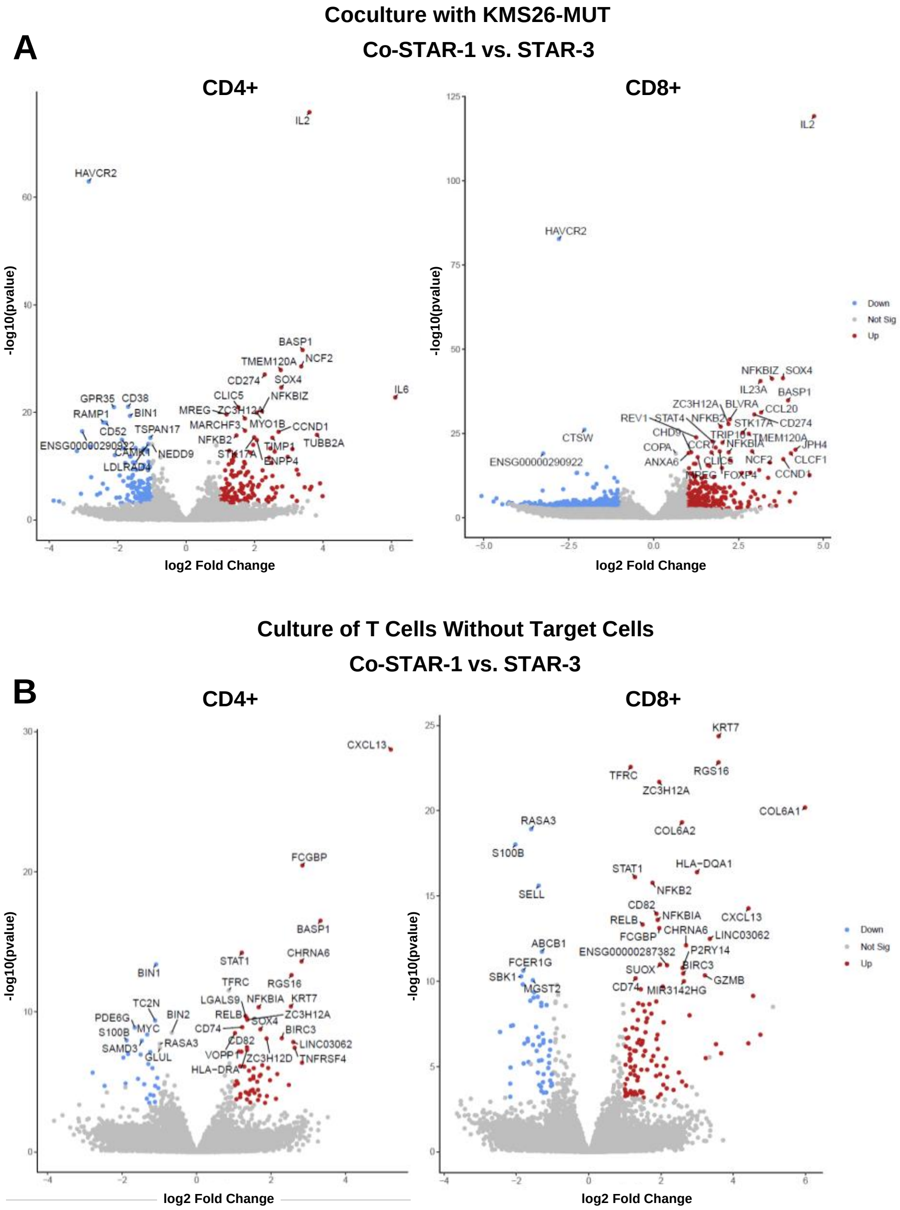

Fig. S13. Volcano plots for Co-STAR-1 T cells compared to STAR-3 T cells.

(A) Genes expressed differentially between Co-STAR-1 and STAR-3 T cells after co-culture with KMS26-MUT cells for 18 hours, with CD4+ T cells displayed on the left and CD8+ T cells on the right. (B) Genes expressed differentially between Co-STAR-1 and STAR-3 after culture without target cells for 18 hours, with CD4+ T cells displayed on the left and CD8+ T cells on the right. Dots are colored red or blue when adjusted p-value is less than 0.05 and the absolute value of the log2 fold change is greater than 1.

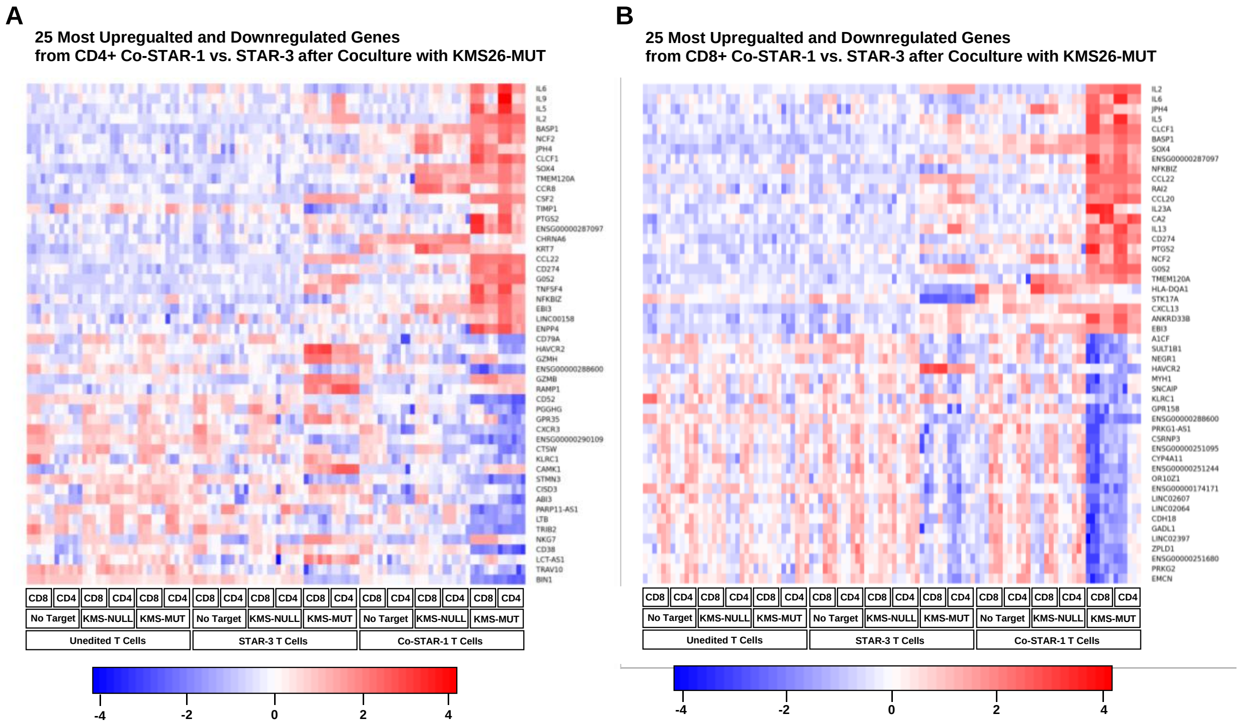

Fig. S14. Heatmaps of differentially expressed Co-STAR-1 T cells.

(A) The 25 most upregulated genes and downregulated genes by log2 fold change between CD4+ Co-STAR-1 and STAR-3 T cells after co-culture with KMS26-MUT cells for 18 hours. (B) The 25 most upregulated genes and downregulated genes by log2 fold change between CD8+ Co-STAR-1 and STAR-3 T cells after co-culture with KMS26-MUT cells for 18 hours. For A and B, differentially expressed genes displayed on heatmaps were filtered for base-mean expression above 10 and scaled by row.

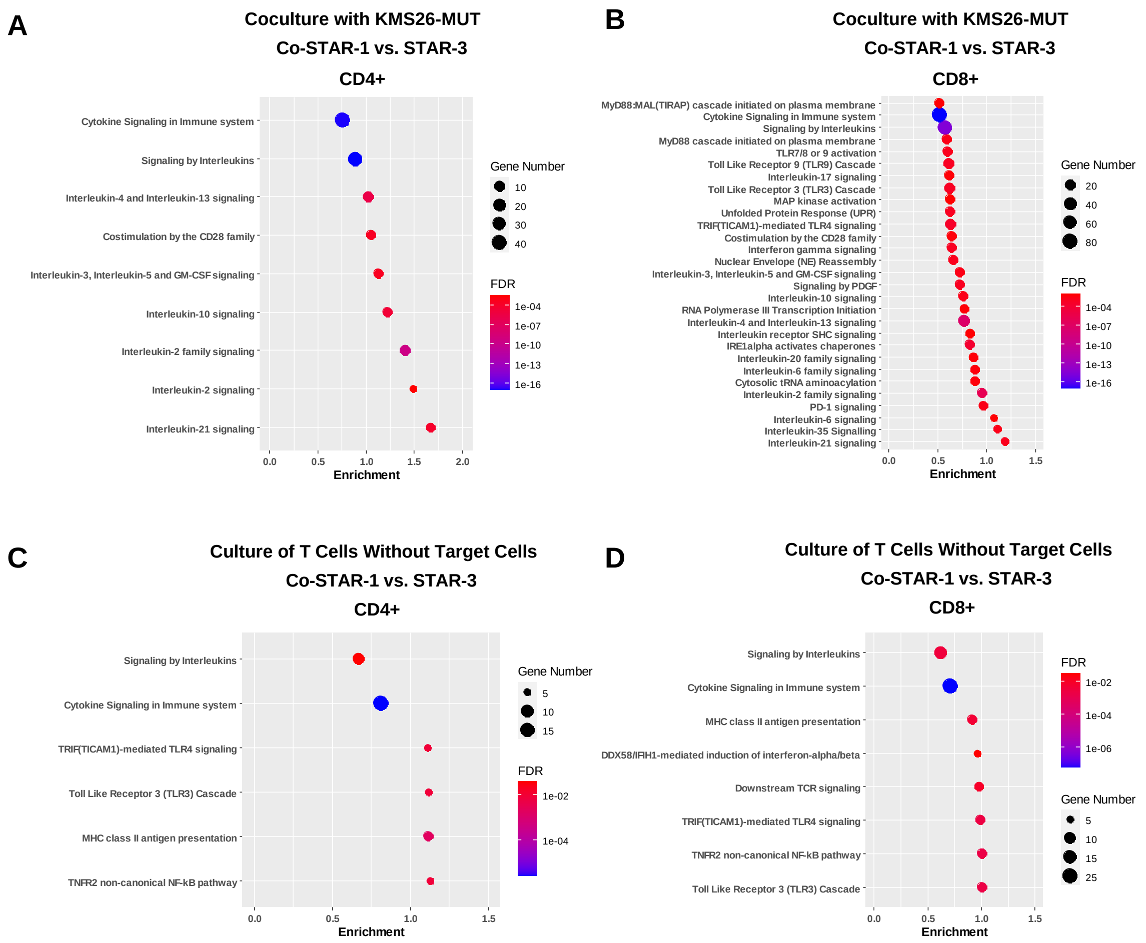

Fig. S15. Pathway analysis of Co-STAR-1 T cells.

(A) STRING analysis of genes upregulated in CD4+ Co-STAR-1 T cells compared to STAR-3 T cells after co-culture with KMS26-MUT cells. (B) STRING analysis of genes upregulated in CD8+ Co-STAR-1 T cells compared to STAR-3 T cells after co-culture with KMS26-MUT cells. (C) STRING analysis of genes upregulated in CD4+ Co-STAR-1 T cells compared to STAR-3 T cells after culture in the absence of target cells. (D) STRING analysis of genes upregulated in CD8+ Co-STAR-1 T cells compared to STAR-3 T cells after culture in the absence of target cells. For A to D, genes input into STRING analysis were identified through DEseq2 with adjusted p-values less than 0.05 and fold change greater than 1.2. No pathways were identified in down-regulated genes.

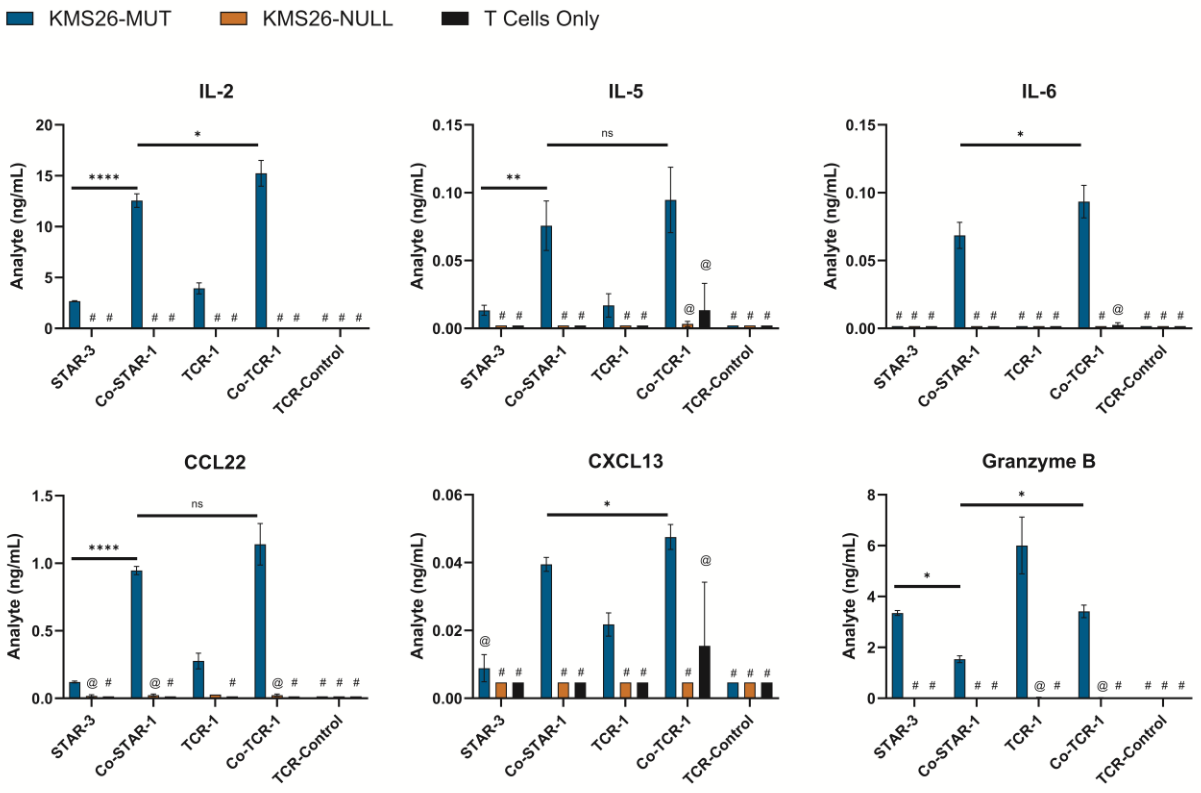

Fig. S16. Markers of Co-STAR activation in vitro after KMS26 isogenic co-culture. Modified T cells (1 x 10^4) were incubated with the KMS26 isogenic cell set at an E:T ratio of 1:5 for 22 hrs in the absence of exogenous IL-2. Conditioned supernatant was analyzed by Luminex for 6 analytes. Data show means ± SD of three technical replicates except for TCR-Control conditions which represent one or two technical replicates. Representative of N = 2 independent experiments. # indicates that all measurements were below the lower limit of detection (LLD) and the LLD is plotted for each replicate. @ indicates that at least one measurement was below LLD and was plotted as the LLD. ****$P < 0.0001$, **$P < 0.01$, *$P < 0.05$, ns (not significant) by one-way ANOVA with Tukey's multiple comparison test for all panels except IL-6 for which an unpaired t-test was used.

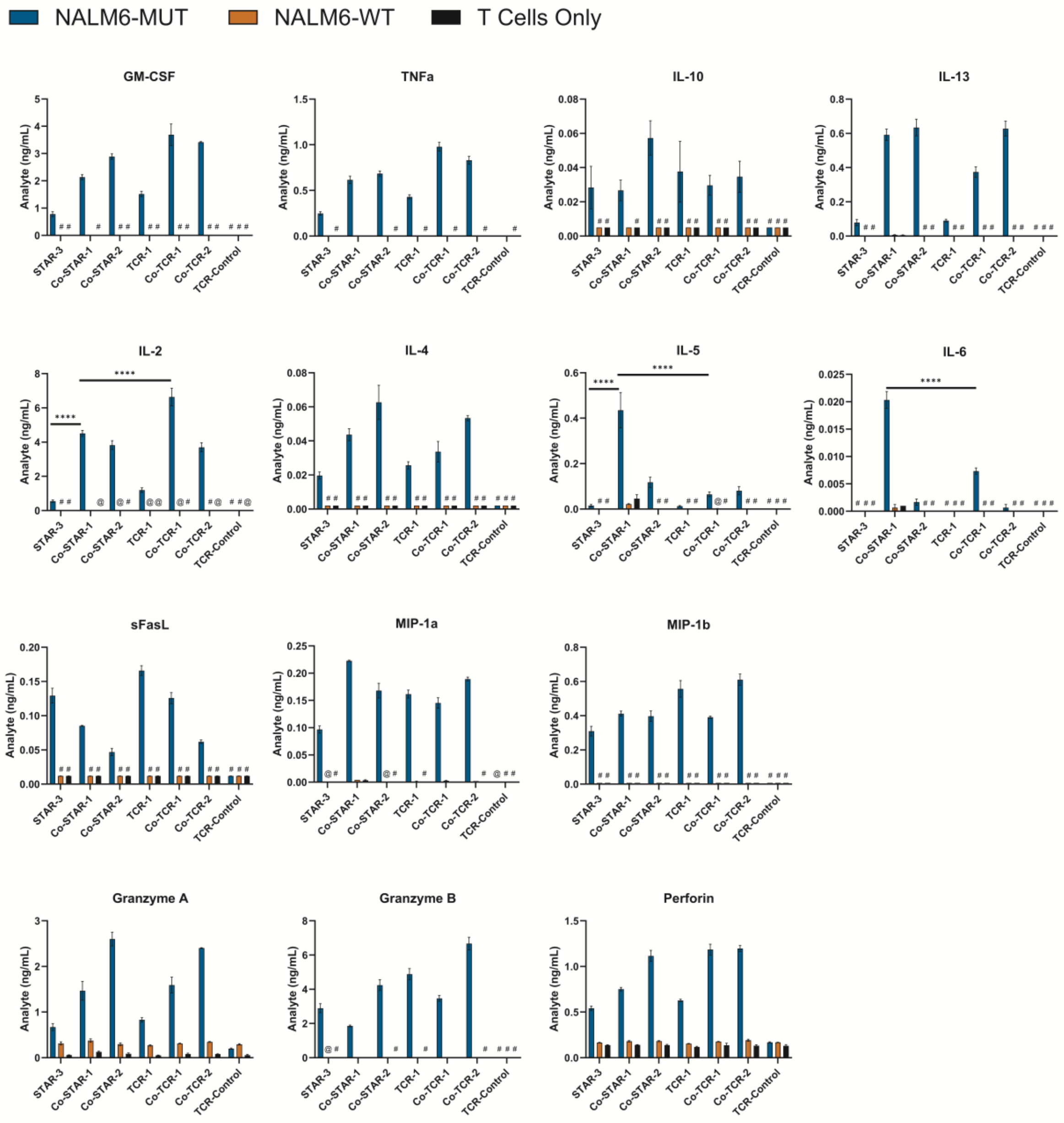

Fig. S17. Markers of Co-STAR activation in vitro after NALM6 isogenic co-culture.

Modified T cells (1 x 10^4) were incubated with the NALM6 isogenic cell set at an E:T ratio of 1:5 for 22 hrs in the absence of exogenous IL-2. Conditioned supernatant was analyzed by Luminex for 15 analytes. Data show means ± SD of three technical replicates. # indicates that all measurements were below the LLD and the LLD is plotted for each replicate. @ indicates that at least one measurement was below LLD and was plotted as the LLD. ****P < 0.0001 by one-way

ANOVA with Tukey's multiple comparison test for all panels when indicated except IL-6 for which an unpaired t-test was used. Data in this figure were from N = 1 experiment.

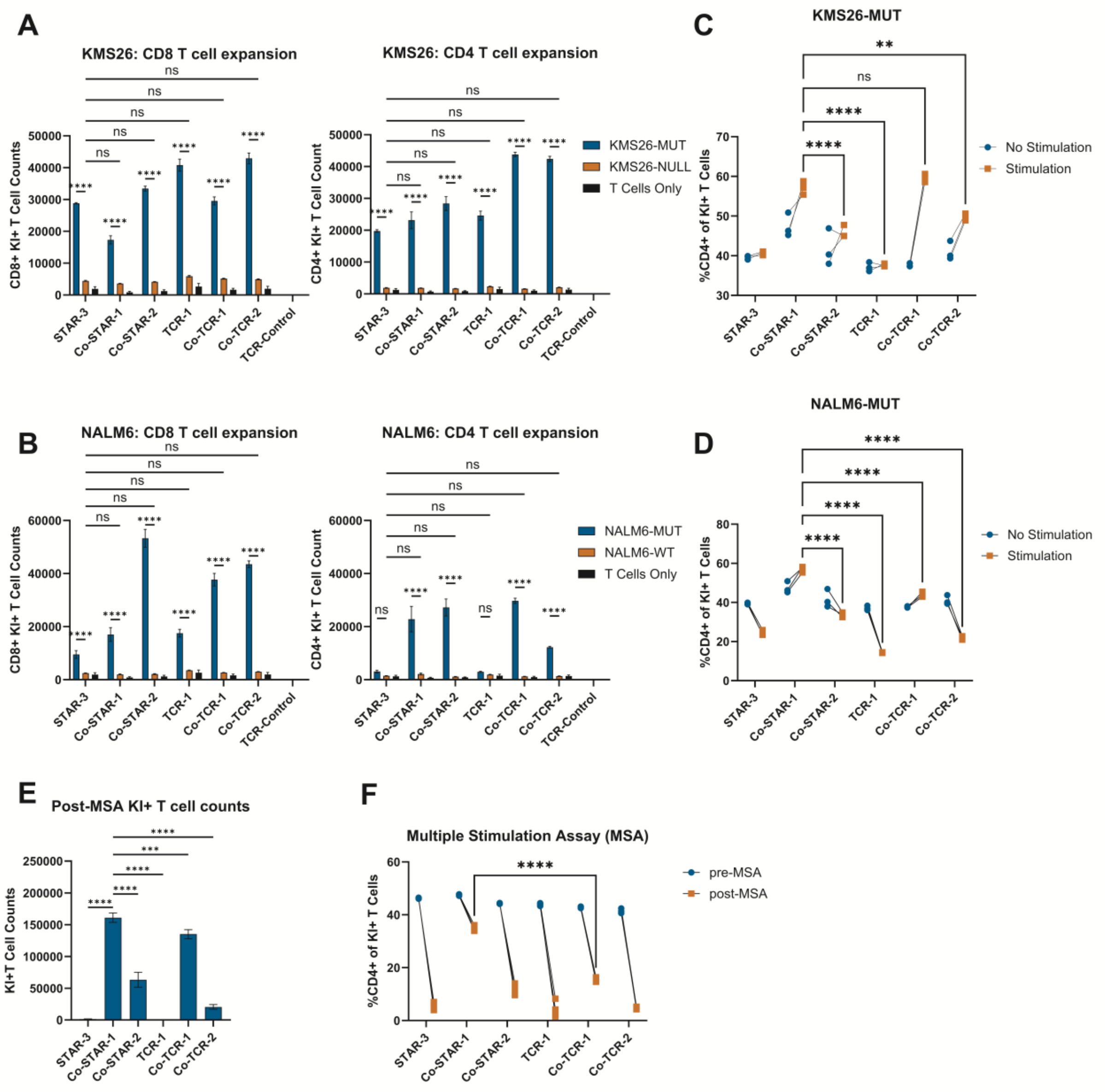

Fig. S18. MyD88-CD40 promotes expansion and persistence of CD4 T cells.

(A) Modified T cells (1 x 10^4) were incubated with the KMS26 isogenic cell set (5 x 10^4) in the presence of exogenous IL-2 for 5 days, after which CD8+ and CD4+ knock-in+ (KI+) T cell numbers were quantified. (B) The same co-culture conditions were used except with the NALM6 isogenic cell set. The same T cell only condition is displayed in both A and B. (C) The percent CD4+ KI+ T cells with and without stimulation were determined after the KMS26 co-culture shown in A. (D) The percent CD4+ KI+ T cells with and without stimulation were determined

after the NALM6 co-culture shown in B. The same T cell only condition was used as reference for panels C and D. (E) Modified T cells (1×10^4) were incubated with NALM6-MUT cells (5×10^4) in the presence of exogenous IL-2. Every 2 days for 12 days, 5×10^4 NALM6-MUT cells in IL-2 media were added to the culture. At the end of 12 days, the number of total KI+ T cells were quantified by flow cytometry. (F) The percent CD4+ KI+ T cells were determined before and after the NALM6 multiple stimulation assay shown in E. In all panels, when applicable data shown are means ± SD of three to four technical replicates. Representative of N = 2 independent experiments. ****$P < 0.0001$, ***$P < 0.001$, **$P < 0.01$, *$P < 0.05$, ns (not significant) by two-way ANOVA with Tukey's multiple comparison test for all panels except C for which a one-way ANOVA with Tukey's multiple comparison was used.

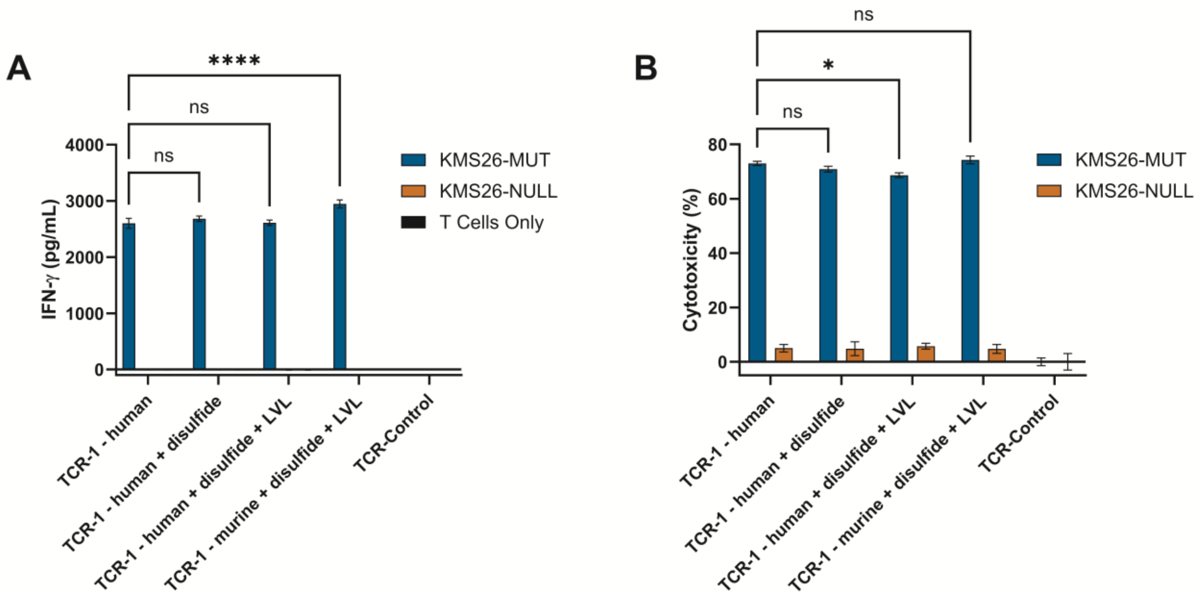

Fig. S19. Comparison of TCR-1 with human vs. modified murine TCRα and TCRβ constant domains.

(A) Modified T cells were cultured with the KMS26 isogenic cell set at an E:T ratio of 1:5 for 21 hrs. "Human" vs. "murine" designates the species of origin of the TCRα and TCRβ constant domains. "Disulfide" indicates the presence of an additional engineered disulfide bond between the TCRα and TCRβ constant domains (*52*). "LVL" indicates the presence of hydrophobic amino acid substitutions in the transmembrane domain of the TCRα chain (*52*). Conditioned supernatant was assayed for IFN-γ by ELISA. (B) The cytotoxicity of modified T cells in the same co-culture was quantified by bioluminescence. Data are shown as means ± SD of three technical replicates, except for the T Cells Only conditions, which are two technical replicates. Data are representative of N = 1 experiment. ****$P < 0.0001$, *$P < 0.05$. ns, not significant, by two-way ANOVA with Tukey's multiple comparison test.
